# Advancing immunotherapy via multiple immune cells co-engagement

**DOI:** 10.3389/fimmu.2026.1783276

**Published:** 2026-02-27

**Authors:** Han Li, Yuxuan Zhang, Qiuyang Wei, Qimeng Sun, Feng Lin, Peng R. Chen

**Affiliations:** 1 Peking-Tsinghua Center for Life Sciences, Peking University, Beijing, China; 2 Synthetic and Functional Biomolecules Center, Beijing National Laboratory for Molecular Sciences, Key Laboratory of Bioorganic Chemistry and Molecular Engineering of Ministry of Education, College of Chemistry and Molecular Engineering, Peking University, Beijing, China

**Keywords:** antitumor immunotherapy, immune cell engager, multiple immune cell co-engagement, multispecific antibody, tumor-immune microenvironment

## Abstract

Immunotherapy has demonstrated remarkable clinical success in a wide range of malignancies, owing to its high specificity and durable therapeutic effects. However, its efficacy is constrained by multiple factors arising from the complex and heterogeneous tumor microenvironment (TME). Strategies capable of simultaneously and synergistically engaging multiple immune cells in TME represents a promising yet challenging frontier. Here we begin with a brief overview of current immune cell engagers harnessing the single immune cell types, such as T, NK cells and other immune cells. We then focus on the next generation of multiple immune cell-type co-engagement immunotherapies, discussing their targets, mechanisms, and therapeutic design. This review outlines both opportunities and hurdles of the multiple immune cell co-engagers, paving the way for more effective antitumor modalities.

## 1 Introduction

The advent of immunotherapy represents one of the most significant milestones in the history of cancer medicine (1). Early interventions focused on single immune checkpoints (2, 3) or mono-lineage immune activation (4, 5), while the efficacy of these treatments is frequently compromised by a multitude of factors, most notably those stemming from the intricate and often immunosuppressive nature of the TME (6). These barriers render traditional immunotherapy insufficient for a vast majority of patients with solid tumors.

To overcome these hurdles, the field is shifting toward a more holistic strategy that enables the simultaneous and synergistic targeting of multiple immune cell types. A potent antitumor response is not the result of a single cell type acting in isolation, but rather a coordinated “immune symphony” (7). For instance, optimal CD8^+^ T cell effector function and response to PD-1 blockade are critically dependent on the crosstalk between T and dendritic cells (DCs) (8, 9), specific niches of myeloid antigen-presenting cells (APCs) (10) as well as costimulatory signals from inflammatory monocytes (11, 12), CD4^+^ T follicular helper (T_FH_) cells and B cells (13). Therefore, therapies that bridge innate and adaptive immunity, orchestrating interactions between T cells, natural killer (NK) cells, and myeloid subsets, hold great promise of converting immunosuppressive microenvironments into inflamed and tumoricidal niches.

Central to this paradigm shift is the concept of multiple immune cell co-engagers. These are defined as multispecific therapeutics that are engineered to simultaneously harness, both physically and functionally, two or more distinct immune cell populations for synergistic tumor eradication, a process that strictly depends on concurrent spatiotemporal engagement rather than on sequential recruitment or indirect cascades. This developments in molecular engineering has birthed a new class of multispecific agents capable of inducing artificial “synapses” between lymphocytes (e.g., T/NK cells) (14) and myeloid cells (e.g., DCs) (15). In this review, we briefly recapitulate the landscape of traditional single immune cell engagers, with a primary focus on the emerging paradigm of multiple immune cell co-engagers. We examine strategies for the co-engagement of both lymphoid and myeloid lineages, delineating their technological platforms, mechanistic underpinnings, and prospective applications in clinical antitumor immunotherapy.

To ensure a comprehensive and rigorous analysis of this rapidly evolving field, literature was identified through systematic searches of PubMed, Web of Science, and ClinicalTrials.gov, covering publications up to January 2026. The search strategy utilized key terms including “T cell engagers”, “NK cell engagers”, “detrintic-T cell engagers”, “NK-T cell engagers”, “bispecific antibody” and “multispecific antibody”. We prioritized English-language, peer-reviewed articles that elucidate mechanistic novelty or demonstrate significant therapeutic efficacy. In synthesizing the evidence, we adopted a hierarchical approach: for single immune cell engagers, priority was given to clinical trial outcomes and regulatory approvals to assess real-world efficacy and safety. Conversely, for emerging strategies multiple immune cell co-engagers, where clinical data are nascent, we relied on high-quality preclinical studies (including *in vivo* solid tumor models) to highlight architectural innovations and mechanistic potential.

## 2 Nowadays: single immune cell-type engagers

The development of immune cell engagers targeting single lineages represents a transformative epoch in clinical oncology, offering potent alternatives to conventional modalities by mechanically redirecting cytotoxic effector cells against malignancies (16). Pioneered by the clinical validation of bispecific T cell engagers, these therapeutics have demonstrated remarkable efficacy, particularly in hematological cancers, by bypassing the major histocompatibility complex (MHC) restrictions to establish synthetic immunological synapses. This section delineates the evolutionary trajectory of these single immune cell-type engagers, then systematically reviews their structural engineering, activation mechanisms, and the iterative refinements designed to surmount current barriers.

### 2.1 The landscape of T cell engagers: from canonical BiTEs to precision-engineered immunotherapeutics

**Figure 1:**
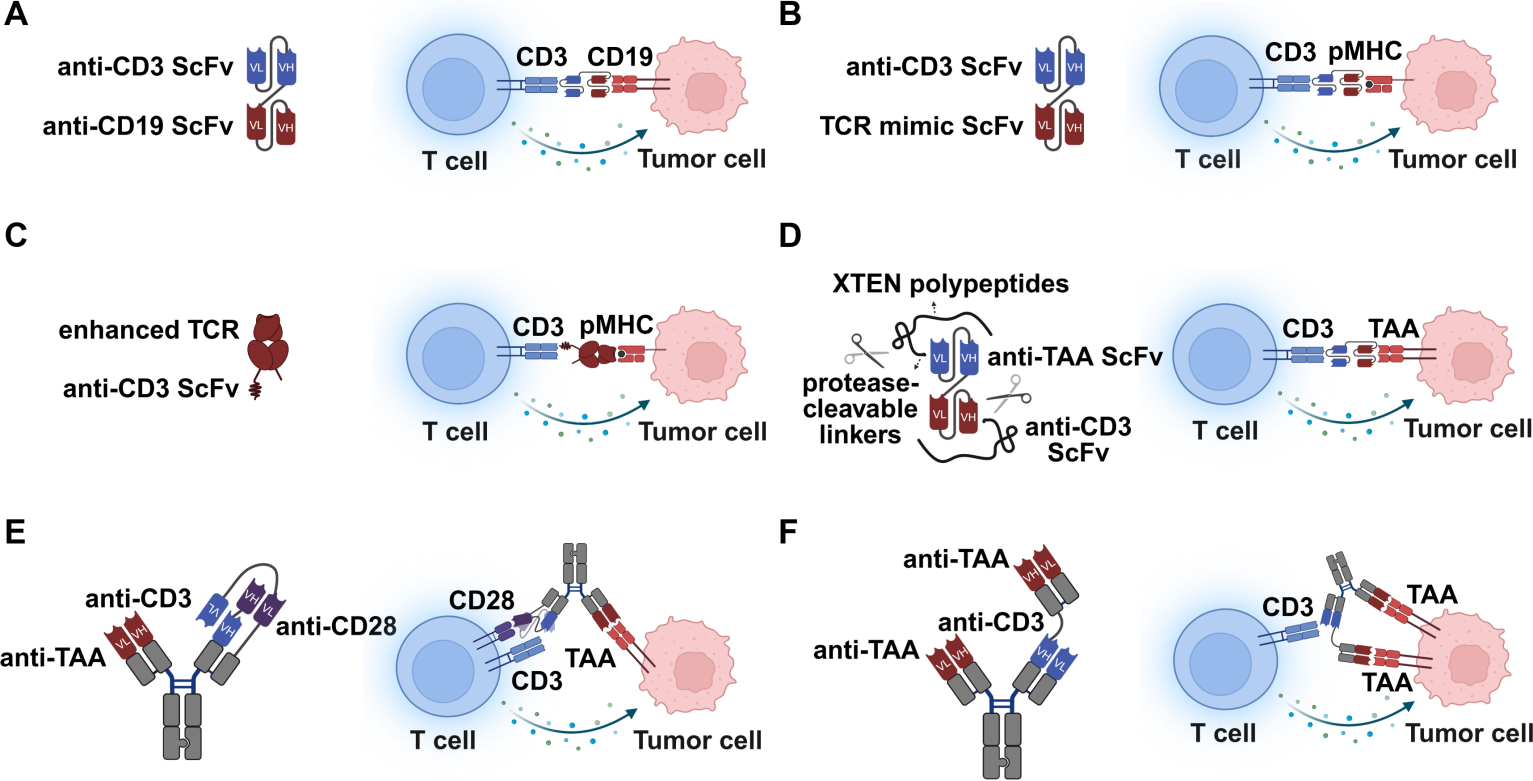
The landscape of T cell engagers. **(A)** Blinatumomab, the first FDA-approved Bispecific T-cell Engager (BiTE), which targets CD19 on B cells and CD3 on T cells. **(B)** T cell engagers targeting peptide-MHC (pMHC) on tumor cells via TCR mimic antibody (TCRm), enabling the recognition of intracellular antigens. **(C)** Immune-mobilizing monoclonal T cell receptor against cancers (ImmTACs). These constructs employ an engineered, high-affinity soluble TCR to recognize pMHC on tumor cells, linked to an anti-CD3 scFv to trigger T cell-mediated effector functions. **(D)** Precision-activated T cell engagers (probodies). These molecules feature masking peptides tethered via protease-cleavable linkers inhibiting antigen binding in peripheral tissues while selectively restoring therapeutic activities upon cleavage by proteases overexpressed in the TME. **(E)** Trispecific engagers designed for co-stimulation. By simultaneously targeting a tumor antigen, CD3, and a co-stimulatory receptor (e.g., CD28), these constructs provide essential “Signal 2” to prevent T cell anergy. **(F)** Dual-targeting T cell engagers. These molecules bind two distinct epitopes or antigens on the tumor cell surface to increase binding avidity and specificity.

As a cornerstone of modern cancer immunotherapy, T cell engagers (TCEs) have fundamentally reshaped the therapeutic landscape by bypassing the MHC restrictions of natural T cell receptors (TCRs) and redirecting cytotoxic T lymphocytes (CTLs) to eradicate tumor cells. The clinical validation of TCEs was established by the approval of blinatumomab, a first-in-class Bispecific T cell Engager (BiTE) targeting CD19 (**Figure 1A**) (17). This format typically links two single-chain variable fragments (scFv), one binding a tumor-associated antigen (TAA) and the other binding CD3ϵ on the T cell. While highly effective in hematological malignancies, the application of classical BiTEs in solid tumors has been hindered by antigenic scarcity, toxicity profiles and T cell anergy. The developmental trajectory of these bispecific molecules has evolved from simple surface-targeting constructs to sophisticated, precision-activated mechanisms designed to overcome the limitations of solid TME. Representative T cell engagers currently approved and in clinical trials are shown in **Table 1**.

**Table 1:** T cell engagers currently approved and in clinical trials.

Molecule name	Mechanism	T cell arm	Tumor antigen	Clinical stage
Blinatumomab	Classic BiTE	CD3	CD19	Approved
Mosunetuzumab	Classic BiTE	CD3	CD20	Approved
Odronextamab	Classic BiTE	CD3	CD20	Approved (only by EMA)
Epcoritamab	Classic BiTE	CD3	CD20	Approved
Teclistamab	Classic BiTE	CD3	BCMA	Approved
Elranatamab	Classic BiTE	CD3	BCMA	Approved
Linvoseltamab	Classic BiTE	CD3	BCMA	Approved
Talquetamab	Classic BiTE	CD3	GPRC5D	Approved
Tarlatamab	Classic BiTE	CD3	DLL3	Approved (only by FDA)
AZD0486	Classic BiTE	CD3	CD19	Phase II
Cevostamab	Classic BiTE	CD3	FcRH5	Phase II
M701	Classic BiTE	CD3	EpCAM	Phase III
IMA401	Targeting pMHC	CD3	MAGEA4/A8	Phase I
IMA402	Targeting pMHC	CD3	PRAME	Phase II
IMC-F106C	Targeting pMHC	CD3	PRAME	Phase III
Flotetuzumab	Reduced affinity of T cell arm	CD3	CD123	Phase II
MGD024	Reduced affinity of T cell arm	CD3	CD123	Phase I
STA551	Reduced affinity of T cell arm	CD3	CD137	Phase I
JANX007	Probody	CD3	PSMA	Phase I
VIR-5500	Probody	CD3	PSMA	Phase I
VIR-5818	Probody	CD3	HER2	Phase I
JANX008	Probody	CD3	EGFR	Phase I
VIR-5525	Probody	CD3	EGFR	Phase I
BA3182	Probody	CD3	EpCAM	Phase I
Glofitamab	Two tumor cell arms	CD3	2*CD20	Approved
ABBV-383	Two tumor cell arms	CD3	2*BCMA	Phase III
Xaluritamig	Two tumor cell arms	CD3	2*STEAP	Phase III
ISB 2001	Two tumor cell arms	CD3	CD38, BCMA	Phase I
JNJ-79635322	Two tumor cell arms	CD3	BCMA, GPRC5D	Phase III
PRS-343	Alternative T cell arm	CD137	HER2	Phase II
RO7122290	Alternative T cell arm	CD137	CD19	Phase II
RO7227166	Alternative T cell arm	CD137	CD19	Phase II
CB307	Alternative T cell arm	CD137	PSMA	Phase I
SAR442257	Two T cell arms	CD3, CD28	CD38	Phase I
PIT565	Two T cell arms	CD2, CD3	CD19	Phase I

*Unless otherwise specified, “approved” means approved by EMA and FDA.

*Clinical stage is summarized up to January 2026.

*BiTE, bispecific T cell engager; CD3, cluster of differentiation 3; CD19, cluster of differentiation 19; CD20, cluster of differentiation 20; BCMA, B cell maturation antigen; GPRC5D, G protein-coupled receptor class C group 5 member D; EpCAM, epithelial cell adhesion molecule; DLL3, Delta-like ligand 3; FcRH5, Fc receptor like 5; BICR5, Baculoviral inhibitor of apoptosis repeat containing 5; MAGEA4/A8, melanoma-associated antigen A4/A8; PRAME, preferentially expressed antigen melanoma; CD123, cluster of differentiation 123; HER2, human epidermal growth factor receptor 2; PSMA, prostate-specific membrane antigen; CD137, cluster of differentiation 137; EGFR, epithelial growth factor receptor; STEAP, six-transmembrane epithelial antigen of prostate; CD28, cluster of differentiation 28; CD2, cluster of differentiation 2; CD38, cluster of differentiation 38.

#### 2.1.1 Expanding the target space: targeting peptide-MHC complexes engagers

Most surface antigens (e.g., HER2, EGFR) are shared with healthy tissues, leading to dose-limiting “on-target, off-tumor” toxicities (18). Furthermore, the vast majority of oncogenic drivers (e.g., p53, RAS) are intracellular and inaccessible to conventional antibodies (19, 20). To address the “undruggable” nature of these intracellular oncoproteins, the field has advanced toward targeting pMHC complexes (**Figures 1B, C**). This has bifurcated into two distinct structural approaches. (i) TCR Mimic (TCRm) Antibodies (**Figure 1B**). Researchers have utilized phage display and hybridoma technologies to engineer antibodies that mimic the specificity of TCRs (21). For instance, Li et al. highlighted the utility of chimeric HLA tetramers to refine the specificity of these TCRm antibodies, reducing cross-reactivity against the MHC backbone (22). Recent work by Hsiue et al. and Douglass et al. also demonstrated the generation of high-affinity “MANAbodies” (Mutation-Associated NeoAntigen antibodies) in a single-chain diabody (scDb) format (23, 24). These bispecific antibodies can distinguish single amino acid changes in mutant p53 (R175H) or RAS neoantigens presented on HLA, inducing potent T cell killing even at extremely low antigen densities (<10 copies/cell). Furthermore, many researchers utilized *de novo* generative models (e.g., RFdiffusion) to design “minibinders” against specific pMHC targets with high precision, bypassing the limitations of animal immunization or library screening (25–27). (ii) Immune-Mobilizing Monoclonal TCRs Against Cancer (ImmTAC) (**Figure 1C**). An alternative strategy employs affinity-matured soluble TCRs fused to anti-CD3 effectors (28). The landmark approval of tebentafusp for metastatic uveal melanoma, as detailed by Nathan et al., validated this class. Tebentafusp targets the gp100 peptide presented by HLA-A*02:01 with picomolar affinity, significantly exceeding the affinity of natural TCRs (29). This ultra-high affinity allows for the recruitment of T cells to tumors with low antigen expression, though it necessitates rigorous safety profiling to prevent cross-reactivity against healthy tissues.

#### 2.1.2 Mitigating toxicity: precision-activated engagers

Despite the potency of TCEs, the therapeutic window remains a critical bottleneck due to systemic toxicity. To decouple antitumor efficacy from systemic side effects, “masked” TCEs (also named probodies) have been developed (**Figure 1D**). As described by Boustany et al. and Cattaruzza et al., these masking peptides link to the antibody domains via protease-cleavable linkers (30, 31). In the circulation and healthy tissues, the mask prevents binding; however, upon entering the TME, dysregulated proteases cleave the linker, unmasking the TCE and restricting T cell activation specifically to the tumor site. This probody strategy has enabled the safe targeting of ubiquitous antigens like HER2 and EGFR in preclinical models with significantly widened therapeutic index (32).

#### 2.1.3 Overcoming T cell anergy: co-stimulation engagers

Canonical TCEs rely solely on “Signal 1” (TCR/CD3 engagement), which in the absence of “Signal 2” (co-stimulation), can lead to the T cell anergy or activation-induced cell death (AICD). To recapitulate a physiological immunological synapse, novel trispecific antibodies have been engineered to engage a tumor antigen alongside both CD3 and the co-stimulatory receptor CD28 (**Figure 1E**). As demonstrated by Wu et al. and Seung et al., this “dual-signaling” approach significantly enhances T cell proliferation through co-stimulation. Crucially, these constructs lower the threshold for T cell activation, enabling the elimination of tumors with low antigen density that are typically resistant to standard TCEs (33, 34).

#### 2.1.4 Improving selectivity: dual-targeting engagers

To mitigate tumor immune escape caused by antigenic drift and to enhance tumor specificity, the field has advanced toward dual-targeting strategies (**Figure 1F**). This approach employs TCEs designed to bind two epitopes on the same target or two distinct tumor-associated antigens (TAAs). Bacac et al. developed a bivalent Fab domain to a single carcinoembryonic antigen (CEA) and a single Fab domain to CD3, increasing avidity and facilitating the selective lysis of high-expressing tumor cells (35). An alternative approach utilizes the IgM-based bispecific antibody. Baliga et al. introduced a high avidity of IgM-based CD20×CD3 bispecific antibody (IGM-2323) to enhance T cell dependent killing with minimal cytokine release (36). Although dual-targeting engagers binding the same target effectively increase the avidity to tumor cells, it may also increase binding to healthy tissues that have low levels of the target antigen and cause toxicity. To address this, Carretero-Iglesia et al. described ISB 2001, a trispecific antibody targeting BCMA and CD38; and Roskopf et al. described 33-3–19 targeting CD19^+^CD33^+^ leukemia cells (37, 38). These molecules leverage “super-avidity” to induce cytotoxicity against tumor cells with low antigen density using two distinct TAAs that escape mono-targeting agents. While Shen et al. and Tapia-Galisteo et al. illustrated this using logic-gated “AND” designs (TriTCEs or TriTEs) (39, 40). These logic-gated TCEs require simultaneous binding to both antigens to trigger robust T cell activation, thereby sparing healthy tissues that express only one of the antigens. This dual-targeting modality is essential for overcoming intratumoral heterogeneity and preventing relapse due to single-antigen loss.

### 2.2 The development of NK cell engagers: from single-activated NKCEs to multifunctional immunotherapeutics

Besides direct T cell-mediated cytotoxicity, the evolution of cancer immunotherapy has expanded to exploit innate immunity through the development of NK cell engagers (NKCEs) (16, 41). The fundamental premise of NKCEs is to physically bridge the innate immune system and tumor cells, establishing a synthetic immunological synapse that actively redirects cytotoxicity. This architectural innovation is designed to overcome the intrinsic limitations of conventional monoclonal antibodies (mAbs) and early-generation engagers. Traditional approaches often rely on stochastic encounters and passive Fc-mediated antibody-dependent cellular cytotoxicity (ADCC), which are frequently compromised by FcγRIIIA (CD16A) polymorphisms, competition from physiological serum IgG, and the downregulation of activating receptors within the immunosuppressive TME (42, 43). Consequently, the developmental trajectory of NKCEs has evolved from simple surface-targeting constructs to sophisticated, precision-activated mechanisms. To address the progressive challenges identified in early research, NKCEs have evolved through three distinct generations. Representative NK cell engagers currently in clinical trials are shown in **Table 2**.

**Table 2:** NK cell engagers currently in clinical trials.

Molecule name	Mechanism	NK cell arm	Tumor antigen	Cytokine payload	Clinical stage
AFM13	One NK cell receptor	CD16A	CD30	No	Phase II
AFM24	One NK cell receptor	CD16A	EGFR	No	Phase II
AFM28	One NK cell receptor	CD16A	CD123	No	Phase I
DF1001	Two NK cell receptors	NKG2D, CD16A	HER2	No	Phase II
DF9001	Two NK cell receptors	NKG2D, CD16A	EGFR	No	Phase II
DF2001	Two NK cell receptors	NKG2D, CD16A	CD33	No	Phase I
DF3001	Two NK cell receptors	NKG2D, CD16A	BCMA	No	Phase I
IPH64	Two NK cell receptors	NKp46, CD16A	BCMA	No	Phase II
SAR443579	Two NK cell receptors	NKp46, CD16A	CD123	No	Phase II
GTB-3550 TriKE	One NK cell receptor	CD16A	CD33	IL15	Phase II

*CD16A-based designs may also interact with other FcγRIIIa-expressing effector cells if using Fc-mediated antibody-dependent cellular cytotoxicity (ADCC).

*Clinical stage is summarized up to January 2026.

*NK, natural killer; CD16A, cluster of differentiation 16A; CD30, cluster of differentiation 30; EGFR, epithelial growth factor receptor; CD123, cluster of differentiation 123; NKG2D, NK group 2, member D; HER2, human epidermal growth factor receptor 2; CD33, cluster of differentiation 33; BCMA, B cell maturation antigen; NKp46, NK cell receptor 46.

#### 2.2.1 Single activating receptor engagers

**Figure 2:**
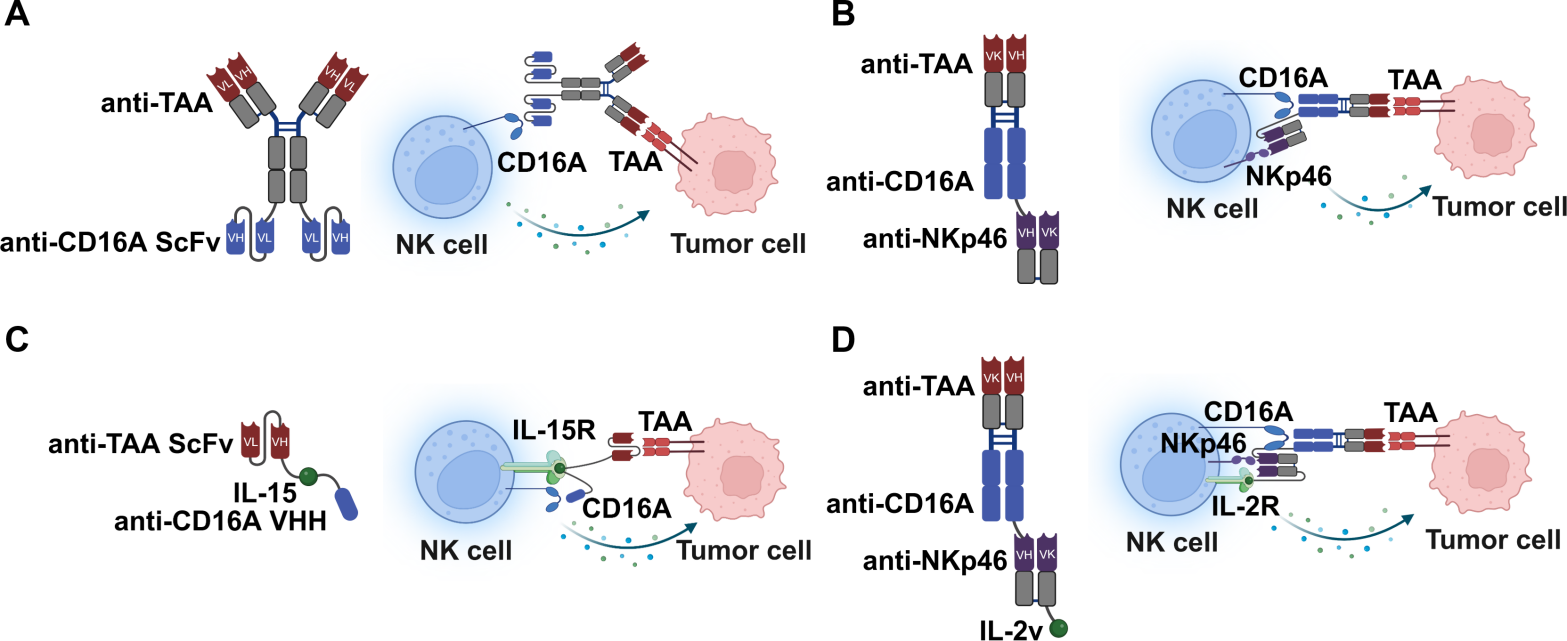
The development of NK cell engagers. **(A)** First-generation engagers targeting the activating receptor CD16A. These molecules are frequently referred to as innate cell engagers (ICEs) as CD16A is shared by other myeloid lineages, including macrophages. **(B)** Second-generation dual-engagers designed to co-engage CD16A alongside a second activating receptor, such as NKp46, to enhance activation stability. **(C)** Cytokine-armored engagers that integrate an activating receptor (CD16A) with an IL-15 to provide essential survival signals. **(D)** Multifunctional cytokine-armored engagers combining dual-receptor co-engagement (CD16A and NKp46) with an engineered IL-2 variant (IL-2v).

The first generation, illustrated in **Figure 2A**, focuses on the robust engagement of FcγRIIIA (CD16A). A paradigmatic example is AFM13, a tetravalent bispecific antibody targeting CD30 and CD16A which has been tested in phase II clinical trial (44). These molecules, often termed Innate Cell Engagers (ICEs), recruit NK cells (and macrophages) to the tumor site. By binding CD16A with high affinity, they trigger the phosphorylation of ITAMs that leads to degranulation and lysis. In addition to AFM13, AFM24 targeting EGFR and targeting CD123 have also been tested in clinical trials. While effective, relying solely on CD16A has vulnerabilities (45). CD16A is prone to proteolytic shedding by ADAM17 upon activation and is frequently downregulated in the immunosuppressive TME, potentially dampening efficacy over time (46).

#### 2.2.2 Dual activating receptor engagers

To overcome the instability of CD16A expression, the second generation incorporates a second activating receptor, such as NKG2D, NKp46 or NKp30 (**Figure 2B**). For instance, NKG2D and CD16A dual activating NKCEs targeting HER2^+^, EGFR^+^, CD33^+^ or BCMA^+^ tumor cells have shown greater activation efficacy and entered clinical trials (47). Nevertheless, persistent engagement of NKG2D by its ligands induces receptor internalization and subsequent NK cell hypo responsiveness (48). Furthermore, the therapeutic potency of these bispecific antibodies is often compromised by the presence of soluble NKG2D ligands released by tumors via proteolytic shedding or exosomal secretion, which act as decoys to intercept the therapeutic agents (49). Gauthier et al. detailed a “trifunctional” NKCE co-engaging NKp46 and CD16A (50). NKp46 is a natural cytotoxicity receptor (NCR) that is constitutively expressed and, crucially, retained on tumor-infiltrating NK cells even when other receptors like NKG2D are downregulated. Expanding the repertoire of dual-targets, Pekar et al. developed a dual activating NKCE targeting NKp30 and CD16A, which shows synergistic cytotoxicity (51, 52). Although dual activating NKCEs induce synergistic activation, leading to full secretory and cytotoxic competence superior to single-receptor engagement, they do not fully address the issue of NK cell persistence in the absence of exogenous cytokines. In clinical, NK cells in patients are often short-lived or functionally exhausted due to cytokine deprivation in the TME.

#### 2.2.3 Cytokine-armored engagers

Addressing the need for survival signals, the third generation integrates stimulatory cytokines, such as IL-15 or IL-2 variant (**Figures 2C, D**). Cytokine-armored engagers with single activating receptor (**Figure 2C**) are exemplified by 16-15-B7-H3 and 16-15–33 GTB-3550, incorporate an IL-15 moiety between the anti-CD16A and anti-TAA domains (53, 54). Mechanistically, this provides a “survival signal” directly at the immune synapse. The most advanced architecture (**Figure 2D**) represents a convergence of previous strategies, targeting dual activating receptors (CD16A^+^ NKp46) while providing an optimized cytokine signal (IL-2 variant) (55). These molecules utilize an IL-2 variant (IL-2v) engineered to lose binding affinity for the high-affinity IL-2Rα (CD25) subunit. By co-engaging NKp46 and CD16A, they ensure potent tumor lysis via synergistic signaling. Simultaneously, the IL-2v provides proliferation signals to effector NK cells (which express IL-2Rβγ) but strictly avoids stimulating CD25^+^ Regulatory T cells (Tregs). This mechanism selectively boosts the effector function while preventing the activation of immuno-suppressive populations.

### 2.3 Other immune cell engagers

Complementing the cytotoxic capabilities of TCEs and NKCEs, the therapeutic arsenal of other immune cell engagers has expanded to encompass the myeloid compartment, which often constitutes the most abundant immune population within the TME. Myeloid lineages possess the distinct versatility to execute tumor clearance via antibody-dependent cellular phagocytosis (ADCP) and to orchestrate adaptive immunity through antigen presentation (56, 57). However, given the propensity of intratumoral myeloid cells to adopt immunosuppressive or pro-tumorigenic phenotypes, the development of myeloid cell engagers aims not merely to redirect effector function, but to fundamentally reprogram the TME (16). This emerging class of therapeutics exploits two primary mechanistic strategies to convert myeloid cells from passive bystanders into potent antitumor effectors.

One strategy is to bridge tumor antigens with activating FcRs on myeloid cells, thereby triggering antibody-dependent cellular phagocytosis (ADCP) and cytotoxicity. To avoid the immunosuppressive inhibitory receptor CD32B, engagers are engineered to selectively bind activating receptors such as CD64 or CD89 (58). For instance, bispecific antibodies targeting CD64 and tumor antigens like HER2 have shown promise in promoting ADCP (59, 60). Furthermore, targeting CD89 via IgA-based or bispecific IgG constructs can potentate neutrophil-mediated tumor killing (61, 62).

Another strategy is to deliver costimulatory signals, such as CD40 agonists specifically to the tumor site for overcoming the immunosuppressive TME. By tethering CD40 activation to tumor antigens (e.g., mesothelin) (63), these molecules promote the activation of APCs and enhance T cell cross-priming while minimizing systemic toxicity associated with broad CD40 agonism (64, 65).

Despite the limited efficacy observed in early trials of other immune cell engagers, the field is advancing toward more sophisticated architectures. Myeloid cell engagers have emerged as a promising therapeutic strategy capable of remodeling the TME by reactivating resident myeloid populations.

## 3 Next generation: multiple immune cell-type co-engagers

Despite the milestones achieved by single-lineage engagers, their therapeutic potential in solid tumors is frequently curtailed by the intricate heterogeneity and profound immunosuppression characteristic of the TME. Recognizing that a potent antitumor response is rarely the result of a single cell type acting in isolation, the simultaneous engagement of multiple immune subsets, bridging adaptive effectors with innate antigen-presenting or cytotoxic cells, holds the promise of overcoming resistance mechanisms and preventing escape variants. Herein, we explore the rising of multiple immune cell-type co-engagers, including their distinctive targets, structural engineering and activation mechanisms, then discuss the advanced technological platforms, from genetic fusions, supramolecular assemblies to chemical conjugations that enable this complex multicellular engagement, paving the way for the next wave of precision immunotherapy.

### 3.1 The rising of T-DC co-engagers: targets, formats, and mechanisms

Accumulating evidence indicates that the durability of antitumor immunity relies critically not merely on T cell presence, but on the quality of T cell priming and rejuvenation mediated by professional antigen-presenting cells, specifically DCs (66, 67). Consequently, T-DC co-engagers have emerged as a transformative therapeutic class designed to recapitulate and enforce the natural immunological synapse within the TME. By leveraging the DC’s intrinsic ability to cross-present antigens and orchestrate adaptive immunity, these co-engagers promise to overcome the limitations of “cold” TME and mitigate the systemic toxicities associated with non-specific immune activation. Here we summarize representative T-DC co-engagers with diversity mechanisms, target selection and molecular architecture.

#### 3.1.1 Agonistic co-stimulation and signal replacement

**Figure 3:**
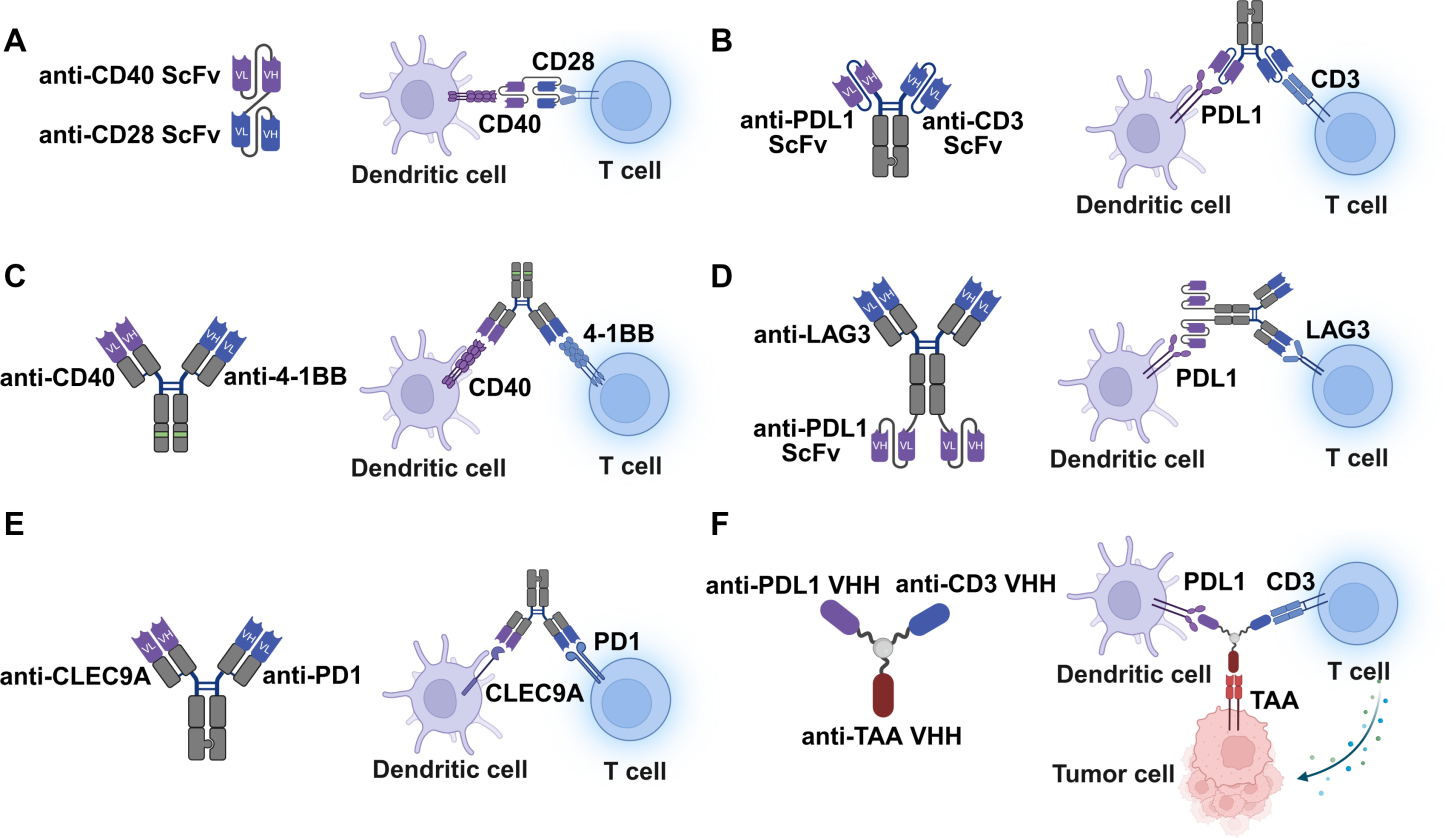
The rising of T cell-DC co-engagers. **(A)** Agonistic co-stimulation. Bispecific antibodies targeting CD28 on T cells and CD40 on DCs to induce simultaneous, reciprocal activation of both cell types. **(B)** Conditional activation. Engagers bridging 4-1BB on T cells and CD40 on DCs relies on physical cross-linking to trigger potent co-stimulation, thereby minimizing off-target activation. **(C)** Checkpoint bridging and rejuvenation. This construct targets CD3 and PD-L1 to block PD-L1 on dendritic cells (but not on tumor cells) and facilitates co-stimulation by B7-1&2, leading to the rejuvenation of CD8^+^ T cells and the durable antigen-specific T-cell responses. **(D)** Dual checkpoint blockade. Co-engager targeting LAG3 on T cell and PDL1 on DC can achieve a dual immune checkpoint blockade effect. **(E)** Bispecific DC-T Cell Engager (BiCE) targeting PD1 on T cell and CLEC9A on DC. CLEC9×PD1 facilitates physical interactions between conventional type I dendritic cells and CD8^+^ PD1^+^ T cells, enhances immune pathways required for aPD-1-mediated response and results with potent antitumor therapeutic activity. **(F)** Multimodal Targeting Chimeras (Multi-TACs). Trispecific agents that simultaneously engage a TAA on tumor cells, CD3 on T cells, and PD-L1 on DCs. This architecture ensures that T-DC crosstalk occurs specifically within the TME.

The earliest iterations of T-DC co-engagers are designed to bypass the reliance on endogenous ligands for co-stimulation. Houtenbos et al. demonstrated a novel bispecific diabody targeting CD40 on DCs and CD28 on T cells (**Figure 3A**) (68). The mechanism here is twofold: agonistic binding to CD40 induces DC maturation and upregulation of B7 molecules, while simultaneous binding to CD28 provides the essential costimulatory signal to T cells. This approach is particularly relevant in acute myeloid leukemia (AML), where leukemic DCs often lack sufficient costimulatory capacity. Building upon this logic, Muik et al. described the DuoBody-CD40×4-1BB, an Fc-inert bispecific antibody (**Figure 3B**) (69). The primary mechanism of action here is conditional agonism, which addresses a critical safety concern associated with monospecific agonists. The stimulation of 4-1BB on T cells and CD40 on APCs occurs only when the bispecific antibody effectively cross-links both cells. This mutual dependency ensures that immune activation is restricted to sites where T cells and APCs interact, thereby widening the therapeutic window and enhancing the priming of tumor-specific CD8^+^ T cells.

#### 3.1.2 Checkpoint bridging and immune rejuvenation

While co-stimulation is vital, the TME requires the simultaneous alleviation of inhibitory signals. A pivotal finding by Liu et al. reshaped our understanding of PDL1 targeting (70). They demonstrated that the therapeutic efficacy of PDL1×CD3 bispecific antibodies (**Figure 3C**) stems not from bridging T cells to tumor cells, but from bridging T cells to PDL1^+^ DCs. Mechanistically, this interaction blocks the PD1-PDL1 inhibitory axis while simultaneously facilitating CD28 co-stimulation via the B7 molecules expressed on the DC surface. This “cis-interaction” on the DC surface leads to the rejuvenation of exhausted CD8^+^ T cells, inducing a durable memory response that conventional T-Tumor engagers fail to generate. Similarly, Sung et al. introduced ABL501, a bispecific antibody targeting LAG3 and PDL1 (**Figure 3D**) (71). This format addresses resistance mechanisms where T cells upregulate alternative checkpoints like LAG3. By simultaneously blocking PDL1 on DCs and LAG3 on T cells, ABL501 enhances T cell activation and, crucially, mitigates the suppressive effects of Tregs, which also express LAG3. These approaches represent a sophisticated integration of checkpoint blockade with physical cell-cell bridging. While the above strategies target DCs broadly, Shapir Itai et al. refined this approach with the Bispecific DC-T Cell Engager (BiCE), which pairs PD-1 targeting on T cells with CLEC9A, a marker exclusive to conventional type 1 dendritic cells (cDC1s) (**Figure 3E**) (72). The primary mechanism here is high-precision spatial orchestration: BiCE physically facilitates the interaction between cDC1s (critical for cross-presentation) and PD1^+^ T cells in draining lymph nodes and the TME This targeted engagement specifically promotes the expansion of progenitor exhausted T cells (T_PEX_), a stem-like population essential for sustained antitumor immunity, which identified as the pivotal cellular targets governing the efficacy of immune checkpoint blockade. Despite these advancements, ensuring that T-DC interactions occur specifically in the vicinity of the tumor remains a challenge.

#### 3.1.3 Multimodal integration and spatial orchestration

To achieve T-DC co-engagement within TME, Lin et al. introduced a breakthrough with Multimodal Targeting Chimeras (Multi-TACs, **Figure 3F**), facilitated by a triple orthogonal linker (T-Linker) chemical conjugation technology (15). The EGFR-CD3-PDL1 Multi-TAC moves beyond bispecific to tri-specific integration, forms a synthetic trimeric cellular complex (Tumor-T-DC) with targeting EGFR on tumor cells for localization, CD3 on T cells for engagement and activation, and PDL1 on DCs for engagement and checkpoint blockade. This spatial orchestration ensures that T cell priming by DCs happens in the immediate presence of tumor antigens, leading to robust cytotoxicity and minimizing off-target effects. The modularity of Multi-TACs allows for the incorporation of diverse immunomodulators, creating a programmable system for “immune management” within the TME.

### 3.2 The emergence of T-other immune cell co-engagers: synergy, remodeling, and modulation

While the specific engagement of DCs is pivotal for rejuvenating adaptive T-cell immunity, the TME hosts a rich repertoire of other potent effector cells. Recognizing this, the field is expanding the co-engager paradigm to orchestrate a more holistic immune response. Emerging strategies now aim to bridge T cells with key components of the innate immune system (such as NK cells, myeloid cells) and regulatory populations (Tregs). This broader class of T-other immune cell co-engagers seeks to synergize the precision of adaptive immunity with the rapid, MHC-independent cytotoxicity of innate effectors, or achieve the remodeling and modulation toward TME, offering a multipronged strategy to overcome tumor heterogeneity and resistance.

#### 3.2.1 Synergy: T-NK cell co-engagers

T cells are potent effectors of adaptive immunity, their efficacy is often limited by tumor heterogeneity, specifically the downregulation of MHC-I molecules. While NK cells, acting as the “first responders” of innate immunity, operate independently of MHC restriction and can eliminate MHC-deficient tumor cells via “missing-self” recognition (73). By simultaneously harnessing these two cell types, co-engagers integrate the power of both T cells in adaptive immunity and NK cells in innate immunity, creating a synergistic attack that prevents tumor immune escape.

**Figure 4:**
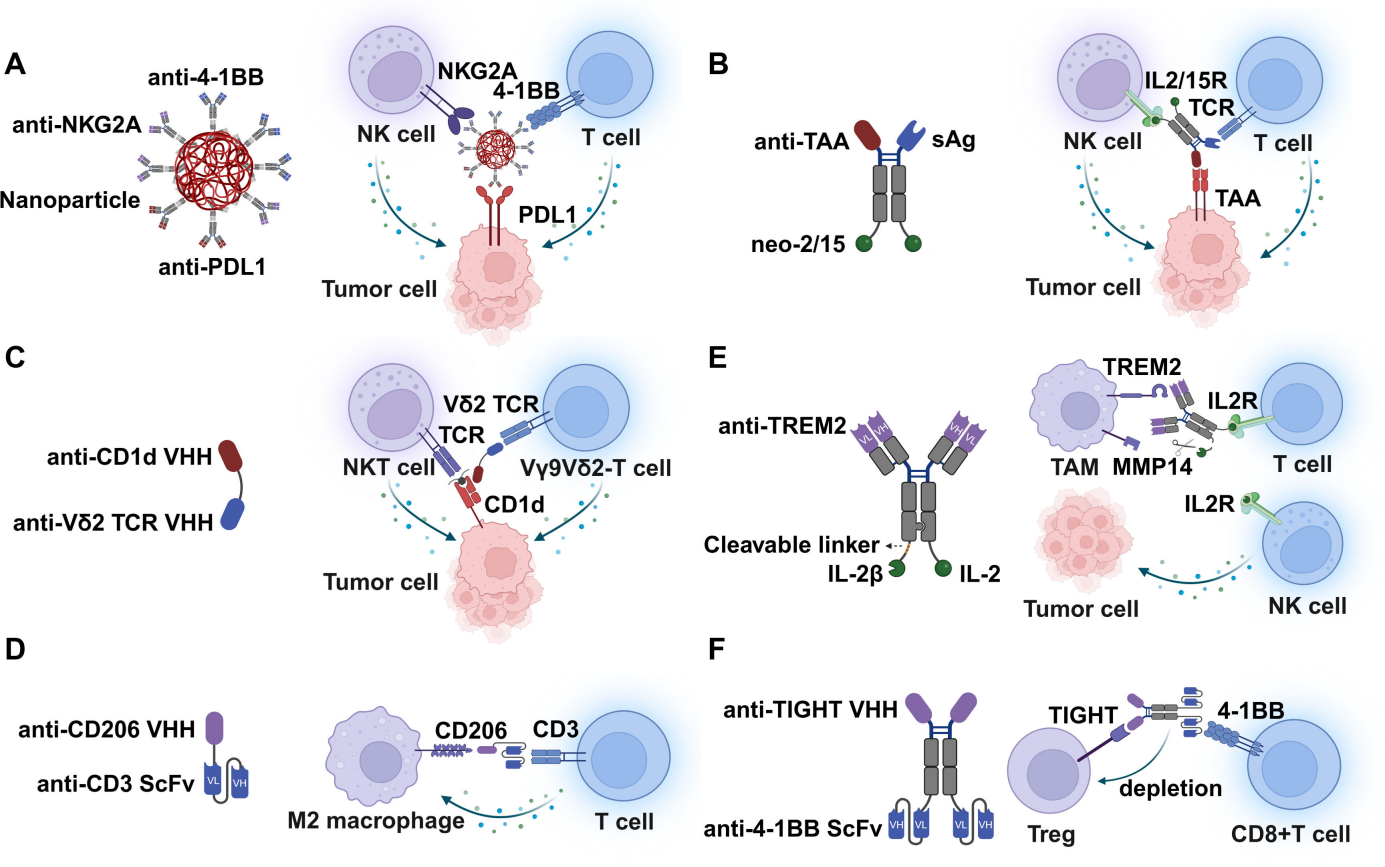
The emergence of various T cell-centered co-engagers. **(A)** T-NK cell co-engagers targeting a TAA on tumor cell, 4-1BB on T cell and NKG2A on NK cell based on nanoparticle platforms. This design coordinates the simultaneous activation of adaptive and innate immunity. **(B)** T-NK cell co-engager via high-affinity superantigen and neo-2/15 targeting tumor cell via TAA. High-affinity superantigens achieve simultaneous activation of T cells and NK cells, while neo-2/15 further enhance T cells and NK cells expansion and survival. **(C)** Vγ9Vδ2 T-NKT cell co-engager via TCR targeting tumor cell via CD1d. CD1d-Vd2 bsTCE selectively engages Vg9Vd2-T and type 1 NKT cells, results in robust antitumor activity and is well tolerated. **(D)** T-M2 macrophage co-engager via CD3 on T cell and CD206 on M2 macrophage. Activated T cells deplete M2 macrophages in the TME. **(E)** Myeloid-targeted immunocytokines and natural killer (NK)-T cell enhancer (MiTEs). MiTEs simultaneously antagonize TREM2 and locally release IL-2 superagonists within tumors, achieving highly efficient synergistic reprogramming of myeloid cells and lymphocytes. **(F)** T-Treg co-engager via 4-1BB on T cell and TIGHT on Treg. Fc-competent TIGIT×4-1BBbispecific antibody exerts potent long-lasting antitumor activity by potentiating CD8^+^ T cell activity and Fcγ receptor- mediated modulation of the TME.

For T-NK cell co-engagers, Ye et al. utilized an albumin/polyester composite nanoparticle (APCN) to construct a Nanoparticle-based Tri-specific Nano-Antibody (Tri-NAb) (74). The nanoparticle surface is coated with anti-Fc antibodies, allowing for the directional immobilization of three distinct monoclonal antibodies (anti-PDL1 targeting tumor cells, anti-4-1BB targeting T cells and anti-NKG2A targeting NK cells) (**Figure 4A**). Mechanistically, it performs three simultaneous functions: it blocks the immune checkpoint PD-L1 on tumor cells; antagonizes the inhibitory receptor NKG2A to unleash cytotoxicity; and agonizes 4-1BB (CD137) to provide potent costimulatory signals. This spatiotemporal synchronization triggers robust activation and proliferation of both CD8^+^ T cells and NK cells, leading to synergistic tumor eradication. After that, Fan et al. optimized the nanoparticle-based platform through FcγR1-serum albumin fusion protein and hydrophobic poly(l-lactide) single-step assembly, achieving the one-step combination of three distinct monoclonal antibodies (anti-PDL1 targeting tumor cells, anti-PD1 targeting T cells and anti-NKG2A targeting NK cells) (75). Furthermore, Yu et al. adopted a yeast-based library and yeast surface display system for directed evolution of Staphylococcal enterotoxin B (SEB), then integrated a tumor-targeting nanobody (e.g., anti-Mesothelin) with an engineered high-affinity superantigen (SEB variant) and a computationally designed cytokine (Neo-2/15) through fusion expression (**Figure 4B**) (14). Unlike conventional bispecific antibodies that rely on anti-CD3 scFv, this construct uses a superantigen to cross-link MHC-II on APCs and specific Vβ chains on the TCR, inducing broad T cell activation. Crucially, the inclusion of the Neo-2/15 cytokine component allows the molecule to simultaneously bind and activate NK cells (which express IL-2/15R). This design promotes the expansion and survival of both T and NK lineages directly at the tumor site, overcoming the limitations of low-affinity superantigens. Additionally, Lameris et al. created a bispecific antibody based on variable domain of heavy chain of heavy chain (VHH) by fusing a CD1d-specific VHH with a Vδ2-TCR-specific VHH (**Figure 4C**) (76). This engager possesses trispecific properties despite being a bispecific molecule. By binding to CD1d on tumor cells, it not only recruits and activates Vγ9Vδ2 T cells but also engages Type 1 NKT cells, which recognize lipid antigens presented by CD1d. This dual recruitment effectively bridges innate-like T cell subsets and NKT cell subsets for tumor control.

#### 3.2.2 Remodeling: T-myeloid cell co-engagers

Myeloid cells, particularly tumor-associated macrophages (TAMs), are another type responders of innate immunity through ADCC and cytokine secretion. However, in solid tumors, TAMs often adopt an immunosuppressive M2-like phenotype, erecting physical and metabolic barriers that exclude T cells (11). Consequently, T-myeloid cell co-engagers are clinically significant because they do not merely target malignant cells; but remodel the stromal architecture to convert “cold” immune-desert tumors into “hot” inflamed environments (77, 78).

For T-myeloid cell co-engagers, Scott et al. engineered bi-valent T cell engagers fusing CD3 scFv targeting T cells and CD206 VHH or folate receptor β scFv targeting M2-like TAMs. They further developed tri-valent T cell engagers, incorporating an additional anti-CD3 or anti-CD28 domain to enhance potency (**Figure 4D**) (79). These agents redirect endogenous T cells to physically engage and lyse M2-like TAMs. This selective depletion of the immunosuppressive stroma creates a pro-inflammatory window, indirectly facilitating antitumor immunity by removing the “brakes” on the TME. Von Locquenghien et al. utilized the distinct characterization of TAMs to remodel TME (80). They developed myeloid-targeted immunocytokines and natural killer (NK)/T cell enhancers (MiTEs), which targeted TREM2 (a myeloid checkpoint on TAMs) and was specifically activated by the tumor-associated protease MMP14 to release IL2 cytokines reacting with IL-2Rβγ on T and NK cells (**Figure 4E**). MiTEs employ a dual-action trans mechanism. First, the antibody antagonizes TREM2, reprogramming TAMs from a suppressive to a pro-inflammatory state. Second, high MMP14 levels on TAMs cleave the linker, unmasking IL-2 locally. This triggers robust proliferation and cytotoxicity in bystander CD8^+^ T cells and NK cells, achieving potent antitumor efficacy with minimal systemic toxicity.

#### 3.2.3 Modulation: T-Treg co-engagers

T-Treg co-engagers function as “ratio-modulators” Their clinical significance lies in simultaneously expanding effector T cells while depleting Tregs, thereby mechanically dismantling the immunosuppressive barrier that limits T cell infiltration and function. Based on the study by Son et al., ABL112 represents a novel Fc-competent bispecific antibody designed to exploit the T-Treg axis (**Figure 4F**) (81). ABL112 is constructed by fusing an anti-TIGIT VHH to the N-terminus of a human IgG1 Fc region, with an anti-4-1BB scFv linked to the C-terminus. It blocks the TIGIT-CD155 interaction to restore T cell function while simultaneously inducing TIGIT-dependent clustering and activation of 4-1BB signaling on CD8^+^ T cells. Since intratumoral Tregs express significantly higher levels of 4-1BB and TIGIT compared to effectors, the Fc-competent design facilitates ADCC mediated by macrophages and NK cells, selectively eliminating Tregs.

### 3.3 Multiple immune cell co-engagement platforms

**Figure 5:**
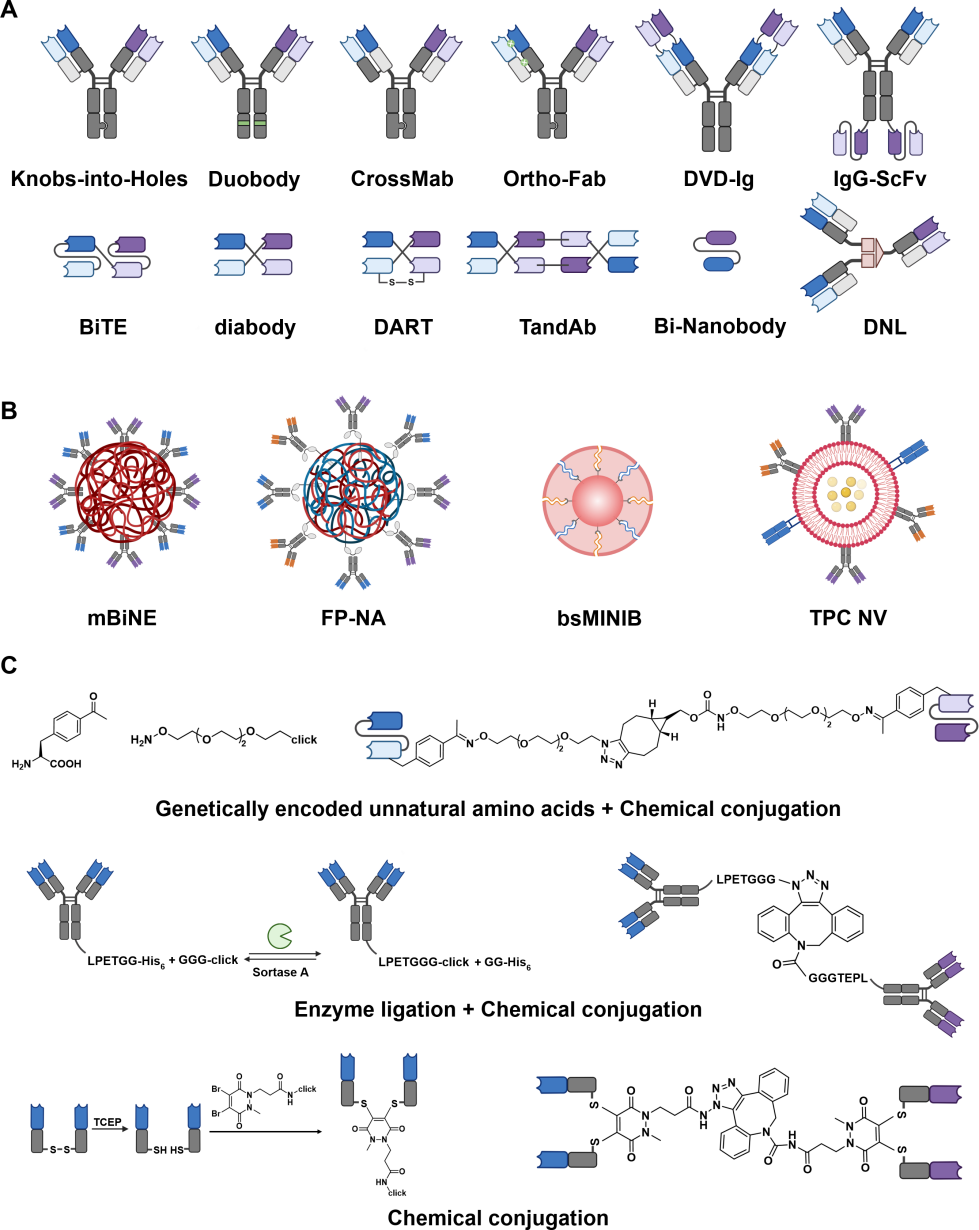
Diverse multiple immune cell co-engagers platforms. **(A)** Genetic fusion. These are categorized into IgG-like formats (including Knobs-into-Holes, DuoBody, CrossMab, Ortho-Fab, DVD-Ig, and IgG-scFv) which retain the Fc region for stability, and non-IgG-like fragment-based formats (including BiTE, Diabody, DART, TandAb, Bi-nanobody, and Dock-and-Lock [DNL]) designed for enhanced tissue penetration. **(B)** Nanomedicines and assemblies. Platforms leveraging nanoparticle scaffolds or vesicles to present multiple ligands. Representative examples include the multivalent bi-specific nanobioconjugate engager (mBiNE), fusion protein/polymer-based nano-adaptors (FP-NA), bispecific molecularly imprinted nanoimmunoblockers (bsMINIB), and TCR nanovesicle antibodies (TCR NV). **(C)** Chemical conjugation strategies. Approaches that employ synthetic chemistry to covalently link distinct binding moieties. These techniques include bioorthogonal reactions utilizing genetically encoded unnatural amino acids (UAAs), site-specific chemo-enzymatic ligation (e.g., via Sortase A), and direct chemical coupling.

The successful translation of multiple immune cell co-engagers from biological concept to clinical reality relies on the underlying engineering platforms designed to construct these complex multispecific agents. As illustrated in **Figure 5**, these platforms can be categorized into three strategies: (i) genetic fusion, which relies on recombinant DNA technology to engineer chimeric protein scaffolds; (ii) nanomedicines or assemblies, which utilize supramolecular chemistry or nanoparticle carriers to present multiple binding ligands; (iii) chemical conjugation, which employs synthetic chemistry, including bio-orthogonal reactions and enzymatic ligation, to covalently link distinct binding moieties.

#### 3.3.1 Genetic fusion

Genetic fusion remains the most prolific approach for generating multispecific co-engagers (**Figure 5A**). These strategies are subdivided into two parts based on the presence or absence of an Fc region: IgG-like formats and non-IgG-like formats (82). Here introduces the representative genetic fusion strategies to construct multispecific co-engagers.

IgG-like formats provide outstanding half-life and stability, but face the primary challenge for preventing random association of heavy and light chains. Knobs-into-Holes (KiH) pioneered by Ridgway et al. engineer steric complementarity into the CH3 domains of the Fc region (83). By introducing a bulky amino acid (T366Y “knob”) into one heavy chain and a smaller residue (Y407T “hole”) into the other, heterodimerization is thermodynamically favored over homodimerization. Furthermore, scientists also use phage display technology to construct a more stable “3 + 1” pattern: with three amino acid mutations (T366S, L368A, and Y407V) form a recessed “holes” type. Duobody utilizes controlled Fab-arm exchange (cFAE). Separately expressed IgG1 molecules containing matched point mutations in the CH3 domain (F405L and K409R) are mixed under mild reducing conditions, driving the efficient recombination of half-molecules into stable bispecific heterodimers (84). To solve light chain mispairing problem, CrossMab swaps CH1 and CL domains within one arm to enforce correct light chain assembly while retaining the KiH Fc structure (85). Similar to CrossMab, Ortho-Fab utilizes orthogonal interfaces engineered via specific mutations at VH/VL or CH1/CL domains to electrostatically or sterically drive the correct pairing of light chains (86). Dual-Variable-Domain Ig (DVD-Ig) and IgG-scFv fuse the other variable domain to the N-terminus or C-terminus, avoiding complex pairing of heterodimers (87, 88).

Non-IgG-like are smaller, fragment-based formats lack an Fc region, offering superior tissue penetration but generally shorter half-lives. They could be directly fused and constructed through simple flexible linkers. BiTE is a tandem scFv format where two scFvs (typically anti-CD3 and antitumor antigen) are linked by a short flexible peptide (17). Diabody uses a short linker (too short for intramolecular pairing) to link a VH domain from one specificity with a VL domain from another on the same polypeptide chain, forcing intermolecular dimerization (89). Dual-Affinity Re-Targeting (DART) is an evolution of diabody (90). DART incorporates a C-terminal disulfide bridge between the two variable domain chains. This covalent linkage significantly enhances stability and reduces the formation of inactive homodimers compared to conventional diabodies. Tandem Diabody (TandAb) is a tetravalent, homodimeric evolution of diabody, formed by the head-to-tail association of two polypeptide chains, each containing four variable domains (91). Bi-nanobody is composed of VHHs which are ligated by flexible linkers (92). Dock-and-Lock (DNL) exploits the natural high-affinity dimerization and docking domain of protein kinase A (PKA) and the anchoring domain of A-kinase anchoring proteins (AKAP) to assemble multivalent complexes (93).

#### 3.3.2 Nanomedicines or assemblies

Besides genetic fusion, nanomedicines or assemblies have emerged as a versatile platform (**Figure 5B**). These supramolecular systems leverage the high surface area-to-volume ratio of nanoparticles to present multiple binding ligands for constructing multispecific co-engagers.

One prominent example is the multivalent bi-specific nanobioconjugate engager (mBiNE) (94). Yuan et al. leveraged carboxyl-functionalized polystyrene nanoparticles as a stable scaffold, employing EDC/NHS chemistry to covalently anchor antibodies via the formation of amide bonds between primary amines on the proteins and carboxyl groups on the nanoparticle surface. By adjusting the molar ratios of the input proteins during the conjugation reaction, the surface density and ratio of proteins can be fine-tuned to optimize avidity without altering the core nanoparticle properties. Ye et al. utilized a more biocompatible material, APCN coated with anti-Fc antibodies to construct a nanoparticle-based Tri-NAb via the directional immobilization of antibodies (74). Furthermore, this team optimized this platform through the single-step assembly of FcγR1-serum albumin fusion protein and hydrophobic poly(l-lactide), which is called the fusion protein/polymer-based nano-adaptor (FP-NA), achieving the one-step combination of antibodies (75). Guan et al. engineered the bispecific molecularly imprinted nanoimmunoblocker (bsMINIB) using molecularly imprinted polymers (MIPs) (95). This platform anchors specific N-terminal epitope peptide of proteins onto a silica nanoparticle substrate via boronate affinity. Subsequently, a silicate layer is polymerized around these templates using functional monomers (e.g., organosilanes). Upon removal of the peptide templates, artificial recognition cavities are left behind in the polymer matrix. This construction creates robust, synthetic receptors (“plastic antibodies”) with high affinity and specificity, avoiding the use of biological antibodies. Leveraging the natural complexity of cell membranes, Li et al. constructed the TCR nanovesicle antibody (TCR NV) using a top-down genetic engineering and membrane extrusion strategy (96). Jurkat T cells are genetically transduced to stably express tumor-specific TCRs alongside proteins scFvs on their plasma membrane. These engineered membranes are then harvested and mechanically extruded to form nanoscale vesicles. This approach preserves the natural topological orientation and transmembrane anchorage of the targeting proteins, while the vesicle lumen serves as a reservoir for loading small-molecule modulators, integrating biologic display with drug delivery in a single construct.

#### 3.3.3 Chemical conjugations

Chemical conjugations represent a highly modular platform for the construction of multiple immune cell co-engagers (97), employing synthetic chemistry to covalently assemble diverse binding moieties and functional payloads, including full-length antibodies, antigen-binding fragments, and immunomodulators (**Figure 5C**).

A representative example of this strategy is the synthesis of IgG-like bispecific antibodies using genetically encoded unnatural amino acids (UAA) (98). Kim et al. demonstrated the site-specific incorporation of p-acetylphenylalanine (pAcF) into the light chains of Fab fragments. These ketone-containing UAAs serve as bioorthogonal handles for conjugation with hydroxylamine-functionalized linkers bearing either an azide or a a bi-cyclo [2.1.0] nonyne (BCN) group. The two functionalized Fabs are then covalently coupled via a copper-free strain-promoted azide-alkyne cycloaddition (SPAAC) reaction. Combining enzymatic precision with synthetic chemistry, Wagner et al. employed a chemo-enzymatic approach utilizing sortase A-mediated transpeptidation to fuse two full-length anti-influenza IgG antibodies at their C-termini (99). Each antibody is first engineered with a sortase recognition motif (LPXTG) and then enzymatically labeled with either an azide or a dibenzocyclooctyne group (DBCO) group. The subsequent SPAAC reaction generates a stable, covalently linked bispecific IgG heterodimer. Expanding the functional complexity of chemical conjugates, Thoreau et al. utilized a pyridazinedione (PD) scaffold to functionally re-bridge the interchain disulfide bonds of Fab fragments while simultaneously introducing bioorthogonal handles (BCN or tetrazine) to construct IgG-like bispecific synthetic antibodies (SynAbs) (100). This team then applied this platform to assemble of a three-protein conjugate site-selectively, with the addition of the third bioorthogonal handles (azide) (101).

**Figure 6:**
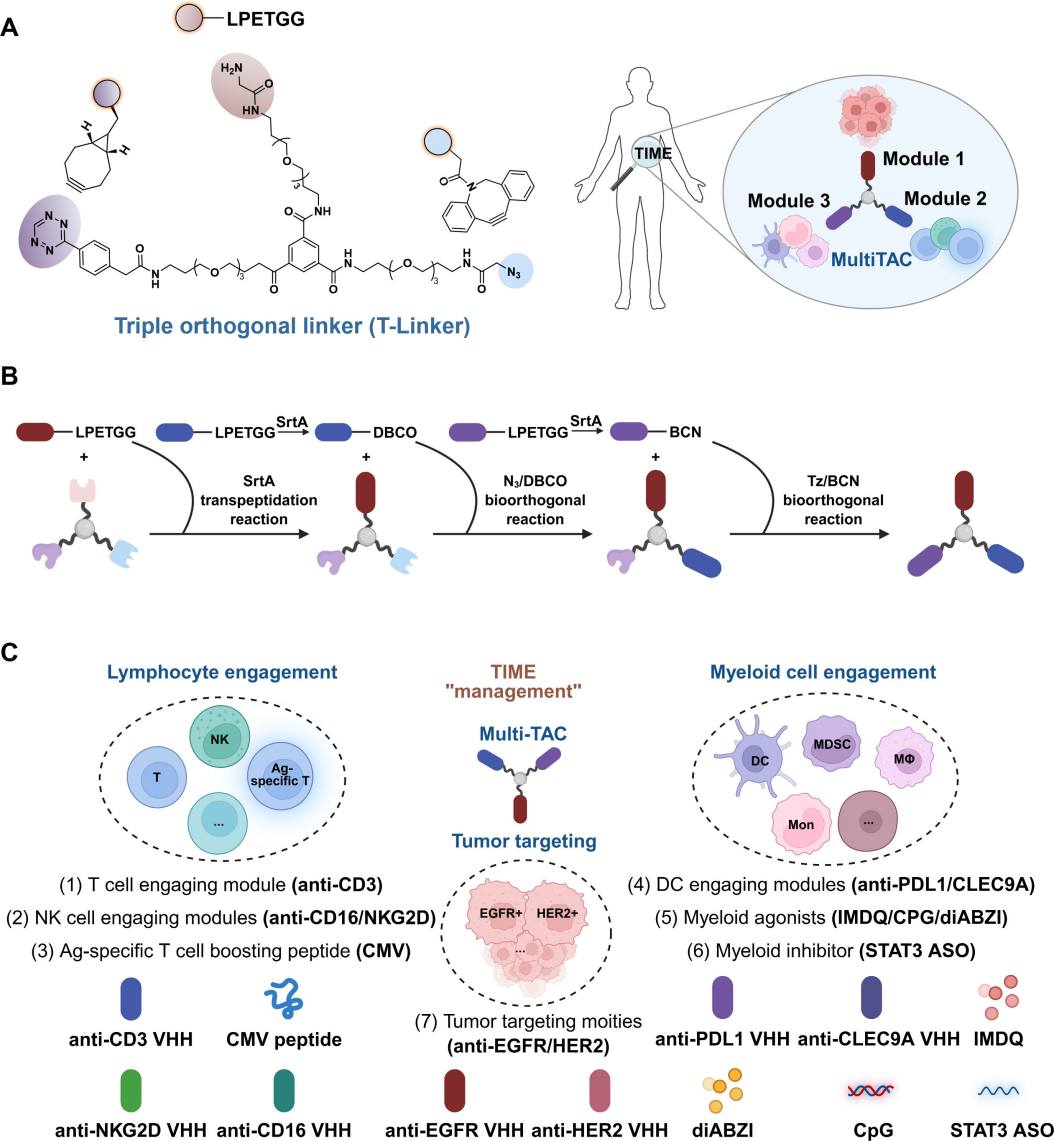
Multi-TAC: a triple orthogonal immune cell co-engager platform for TIME-management. **(A)** Schematic of the Multi-TACs platform. A triple orthogonal linker (T-Linker) contains a single glycine group, an azide group (N_3_) and a tetrazine group (Tz), integrating various therapeutic small molecules and biomolecules as multimodal targeting chimeras (Multi-TACs) through three mutually orthogonal chemical reactions to target the TIME. **(B)** Synthetic workflow. Three different therapeutic modules are first ligated with the T-Linker, a dibenzocyclooctyne group (DBCO) or a bi-cyclo [2.1.0] nonyne group (BCN) using sortase A (SrtA) transpeptidation reaction, and subsequently assembled onto the scaffold through N_3_/DBCO and Tz/BCN bioorthogonal reactions. **(C)** Modular versatility. Multi-TACs are capable of integrating various therapeutic small molecules and biomolecules including proteins, peptides, small molecules and nucleic acids, allowing for the simultaneous engagement of diversity immune cell types including lymphocytes and myeloid cells.

As an advancement in chemical conjugation strategies, a highly modular and programmable platform (Multimodal Targeting Chimera, Multi-TAC) developed by Lin et al. leverages a proprietary triple orthogonal linker (T-Linker) technology (**Figure 6A**) (15). This small-molecule scaffold incorporates a mono-glycine motif, an azide group, and a tetrazine group, facilitating the sequential assembly of three distinct therapeutic modules via sortase A-mediated transpeptidation, strain-promoted azide-alkyne cycloaddition (SPAAC) and inverse electron-demand Diels-Alder (IEDDA) (**Figure 6B**). Functionally, Multi-TACs are engineered to spatially orchestrate multiple immune pathways; thereby coordinating lymphocyte cell with myeloid cell activation with specifically within the tumor microenvironment (**Figure 6C**). Nevertheless, the practical application of this platform faces certain limitations. The reliance on multistep orthogonal reactions necessitates intricate chemical synthesis and purification processes. Furthermore, the *in vivo* pharmacokinetics of these synthetic constructs have not been exhaustively characterized. Despite these challenges, Multi-TACs offer a modular and programmable strategy for the rational design of multispecific agents, representing a promising avenue for future immunotherapeutic development.

## 4 Concluding remarks

For decades, immune cell engagers have primarily focused on engaging a single type of immune cell, most commonly T cells or NK cells. While clinical success has already been achieved with T cell-engaging agents in hematological malignancies, their efficacy in solid tumor is often hindered due to immunosuppressive barriers within the TME. The diversification toward multiple immune cell co-engagers, which encompass innate effectors and other immune subsets, marking a pivotal shift from simple cytotoxicity to comprehensive reprogramming of the TME. This strategy represents a conceptual leap forward, moving beyond the basic “bridging” function of traditional bispecific antibodies toward orchestrating a coordinated “immune symphony.”

By physically and functionally linking adaptive effectors (such as T cells) with innate immune drivers (including NK cells, DCs, and myeloid subsets), these novel modalities address key limitations of single-lineage engagers. Conventional T cell engagers (TCEs), while potent, are frequently compromised by the scarcity of tumor-infiltrating lymphocytes in “cold” tumors, the development of T cell exhaustion and tumor immune escape via antigen downregulation or MHC-I loss. Similarly, the efficacy of NK cell engagers (NKCEs) is often curtailed by the short lifespan of NK cells and their dependence on exogenous cytokines for persistence. While through revitalizing T cell priming via T-DC crosstalk, enhancing synergistic cytotoxicity via T-NK engagement, or remodeling the stromal architecture through T-myeloid cell interactions, this multi-pronged approach provides a robust mechanism to convert immunologically “cold” tumors into inflamed, tumoricidal niches. Nevertheless, each subclass of these co-engagers encounters distinct biological hurdles that must be overcome to achieve optimal efficacy. T-DC co-engagers face the risk of inducing systemic autoimmunity if potent agonists are not strictly confined to the tumor bed. T-NK co-engagers yet are challenged by the distinct cytokine requirements of NK cells, which often fail to persist without exogenous support. T-Myeloid engagers may trigger a “cytokine storm” or failing to fully reverse the M2-suppressive phenotype remains high. Similarly, T-Treg co-engagers face the precarious balance of relieving tumor immunosuppression without breaking peripheral tolerance and causing autoimmune adverse events in healthy tissues.

Besides, the translation of these complex multispecific agents from concept to clinical reality is also restricted by immature technology platforms. Genetic fusion, while versatile, often grapple with protein misfolding and chain mispairing. Nanomedicines and assemblies provide a versatile platform for multivalent presentation and co-delivery, yet can suffer from heterogeneity and potential formulation issues. Chemical conjugations offer modularity, but may involve complex synthesis and purification steps. Despite these advancements, there remains a critical need for a platform that seamlessly integrates the best features of these approaches: modularity, programmability, defined stoichiometry, and superior tumor penetration, while overcoming their respective drawbacks.

Looking forward, despite the biological hurdles and engineering complexities discussed above, the trajectory of multiple immune cell co-engagement remains exceptionally promising, representing a paradigm shift from linear immune signaling to network-based orchestration. The evolution of this field will be defined by the convergence of rational molecular design, chemical biology, and artificial intelligence to develop more clinical promising therapeutics. Clinically, the next generation of therapeutics will likely transition from static multispecific constructs to dynamic, logic-gated systems (e.g., protease-activated probodies) that rely on TME-specific triggers to unlock activity. This strategy ensures immune activation occurs strictly within the tumor bed, thereby decoupling efficacy from systemic toxicity. Furthermore, the successful translation of these agents will depend on moving away from a “one-size-fits-all” approach toward precision stratification, utilizing biomarkers to identify patient subgroups with the requisite immune topography, and deploying synergistic combinations with checkpoint inhibitors or adoptive cell therapies. Ultimately, by harnessing the collective and coordinated power of the distinct immune subsets, multiple immune cell co-engagement holds the potential to dismantle the barriers of tumor heterogeneity and resistance, paving the way for more durable and curative outcomes in the fight against cancer.

## References

[1] Ikeda H . Cancer immunotherapy in progress-an overview of the past 130 years. Int Immunol. (2025) 37:253–60. doi: 10.1093/intimm/dxaf002, PMID: 39792088 PMC11975553

[2] Hodi FS O’Day SJ McDermott DF Weber RW Sosman JA Haanen JB . Improved survival with ipilimumab in patients with metastatic melanoma. N Engl J Med. (2010) 363:711–23. doi: 10.1056/NEJMoa1003466, PMID: 20525992 PMC3549297

[3] Topalian SL Hodi FS Brahmer JR Gettinger SN Smith DC McDermott DF . Safety, activity, and immune correlates of anti-PD-1 antibody in cancer. N Engl J Med. (2012) 366:2443–54. doi: 10.1056/NEJMoa1200690, PMID: 22658127 PMC3544539

[4] Rabik CA Wang S Chadda R Przepiorka D Vallejo J Jiang X . FDA approval summary: Blinatumomab for the treatment of B-cell precursor acute lymphoblastic leukemia in the consolidation phase of multiphase chemotherapy. Clin Cancer Res. (2025) 31:4230–8. doi: 10.1158/1078-0432.Ccr-25-1034, PMID: 40828516 PMC12367060

[5] Neelapu SS Locke FL Bartlett NL Lekakis LJ Miklos DB Jacobson CA . Axicabtagene Ciloleucel CAR T-Cell therapy in refractory large B-cell lymphoma. N Engl J Med. (2017) 377:2531–44. doi: 10.1056/NEJMoa1707447, PMID: 29226797 PMC5882485

[6] de Visser KE Joyce JA . The evolving tumor microenvironment: From cancer initiation to metastatic outgrowth. Cancer Cell. (2023) 41:374–403. doi: 10.1016/j.ccell.2023.02.016, PMID: 36917948

[7] Espinosa-Carrasco G Chiu E Scrivo A Zumbo P Dave A Betel D . Intratumoral immune triads are required for immunotherapy-mediated elimination of solid tumors. Cancer Cell. (2024) 42:1202–16.e8. doi: 10.1016/j.ccell.2024.05.025, PMID: 38906155 PMC11413804

[8] Garris CS Arlauckas SP Kohler RH Trefny MP Garren S Piot C . Successful anti-PD-1 cancer immunotherapy requires T cell-Dendritic Cell crosstalk involving the cytokines IFN-γ and IL-12. Immunity. (2018) 49:1148–61.e7. doi: 10.1016/j.immuni.2018.09.024, PMID: 30552023 PMC6301092

[9] Lei X de Groot DC Welters MJP de Wit T Schrama E van Eenennaam H . CD4(+) T cells produce IFN-I to license cDC1s for induction of cytotoxic T-cell activity in human tumors. Cell Mol Immunol. (2024) 21:374–92. doi: 10.1038/s41423-024-01133-1, PMID: 38383773 PMC10978876

[10] Duraiswamy J Turrini R Minasyan A Barras D Crespo I Grimm AJ . Myeloid antigen-presenting cell niches sustain antitumor T cells and license PD-1 blockade via CD28 costimulation. Cancer Cell. (2021) 39:1623–42.e20. doi: 10.1016/j.ccell.2021.10.008, PMID: 34739845 PMC8861565

[11] Elewaut A Estivill G Bayerl F Castillon L Novatchkova M Pottendorfer E . Cancer cells impair monocyte-mediated T cell stimulation to evade immunity. Nature. (2025) 637:716–25. doi: 10.1038/s41586-024-08257-4, PMID: 39604727 PMC7617236

[12] van Elsas MJ Middelburg J Labrie C Roelands J Schaap G Sluijter M . Immunotherapy-activated T cells recruit and skew late-stage activated M1-like macrophages that are critical for therapeutic efficacy. Cancer Cell. (2024) 42:1032–50.e10. doi: 10.1016/j.ccell.2024.04.011, PMID: 38759656

[13] Cui C Wang J Fagerberg E Chen PM Connolly KA Damo M . Neoantigen-driven B cell and CD4 T follicular helper cell collaboration promotes anti-tumor CD8 T cell responses. Cell. (2021) 184:6101–18.e13. doi: 10.1016/j.cell.2021.11.007, PMID: 34852236 PMC8671355

[14] Yu YA Lien WJ Lin WC Pan YC Huang SW Mou CY . High-affinity superantigen-based trifunctional immune cell engager synergizes NK and T cell activation for tumor suppression. Adv Sci (Weinh). (2024) 11:e2310204. doi: 10.1002/advs.202310204, PMID: 38937984 PMC11434130

[15] Lin F Yin S Zhang Z Yu Y Fang H Liang Z . Multimodal targeting chimeras enable integrated immunotherapy leveraging tumor-immune microenvironment. Cell. (2024) 187:7470–91.e32. doi: 10.1016/j.cell.2024.10.016, PMID: 39504957

[16] Fenis A Demaria O Gauthier L Vivier E Narni-Mancinelli E . New immune cell engagers for cancer immunotherapy. Nat Rev Immunol. (2024) 24:471–86. doi: 10.1038/s41577-023-00982-7, PMID: 38273127

[17] Przepiorka D Ko CW Deisseroth A Yancey CL Candau-Chacon R Chiu HJ . FDA approval: blinatumomab. Clin Cancer Res. (2015) 21:4035–9. doi: 10.1158/1078-0432.Ccr-15-0612, PMID: 26374073

[18] Duro-Sánchez S Alonso MR Arribas J . Immunotherapies against HER2-positive breast cancer. Cancers (Basel). (2023) 15:1069–92. doi: 10.3390/cancers15041069, PMID: 36831412 PMC9954045

[19] Chan A Tsourkas A . Intracellular protein delivery: Approaches, challenges, and clinical applications. BME Front. (2024) 5:0035–64. doi: 10.34133/bmef.0035, PMID: 38282957 PMC10809898

[20] Sorolla A Wang E Golden E Duffy C Henriques ST Redfern AD . Precision medicine by designer interference peptides: Applications in oncology and molecular therapeutics. Oncogene. (2020) 39:1167–84. doi: 10.1038/s41388-019-1056-3, PMID: 31636382 PMC7002299

[21] Chang AY Gejman RS Brea EJ Oh CY Mathias MD Pankov D . Opportunities and challenges for TCR mimic antibodies in cancer therapy. Expert Opin Biol Ther. (2016) 16:979–87. doi: 10.1080/14712598.2016.1176138, PMID: 27094818 PMC4936943

[22] Li D Bentley C Yates J Salimi M Greig J Wiblin S . Engineering chimeric human and mouse major histocompatibility complex (MHC) class I tetramers for the production of T-cell receptor (TCR) mimic antibodies. PloS One. (2017) 12:e0176642. doi: 10.1371/journal.pone.0176642, PMID: 28448627 PMC5407768

[23] Hsiue EH Wright KM Douglass J Hwang MS Mog BJ Pearlman AH . Targeting a neoantigen derived from a common TP53 mutation. Science. (2021) 371:eabc8697. doi: 10.1126/science.abc8697, PMID: 33649166 PMC8208645

[24] Douglass J Hsiue EH-C Mog BJ Hwang MS DiNapoli SR Pearlman AH . Bispecific antibodies targeting mutant RAS neoantigens. Sci Immunol. (2021) 6:eabd5515. doi: 10.1126/sciimmunol.abd5515, PMID: 33649101 PMC8141259

[25] Liu B Greenwood NF Bonzanini JE Motmaen A Meyerberg J Dao T . Design of high-specificity binders for peptide-MHC-I complexes. Science. (2025) 389:386–91. doi: 10.1126/science.adv0185, PMID: 40705892 PMC13077772

[26] Johansen KH Wolff DS Scapolo B Fernández-Quintero ML Risager Christensen C Loeffler JR . *De novo*-designed pMHC binders facilitate T cell-mediated cytotoxicity toward cancer cells. Science. (2025) 389:380–5. doi: 10.1126/science.adv0422, PMID: 40705893

[27] Householder KD Xiang X Jude KM Deng A Obenaus M Zhao Y . *De novo* design and structure of a peptide-centric TCR mimic binding module. Science. (2025) 389:375–9. doi: 10.1126/science.adv3813, PMID: 40705894 PMC12313176

[28] Zareie P Szeto C Farenc C Gunasinghe SD Kolawole EM Nguyen A . Canonical T cell receptor docking on peptide-MHC is essential for T cell signaling. Science. (2021) 372:eabe9124. doi: 10.1126/science.abe9124, PMID: 34083463

[29] Nathan P Hassel JC Rutkowski P Baurain JF Butler MO Schlaak M . Overall survival benefit with Tebentafusp in metastatic uveal melanoma. N Engl J Med. (2021) 385:1196–206. doi: 10.1056/NEJMoa2103485, PMID: 34551229

[30] Boustany LM LaPorte SL Wong L White C Vinod V Shen J . A probody T cell-engaging bispecific antibody targeting EGFR and CD3 inhibits colon cancer growth with limited toxicity. Cancer Res. (2022) 82:4288–98. doi: 10.1158/0008-5472.Can-21-2483, PMID: 36112781 PMC9664135

[31] Cattaruzza F Nazeer A To M Hammond M Koski C Liu LY . Precision-activated T-cell engagers targeting HER2 or EGFR and CD3 mitigate on-target, off-tumor toxicity for immunotherapy in solid tumors. Nat Cancer. (2023) 4:485–501. doi: 10.1038/s43018-023-00536-9, PMID: 36997747 PMC10132983

[32] LaPorte SL Hostetter DR Wong L Razo J Diep L White C . Abstract A203: CD3-EGFR bispecific Probody™ therapeutics induced tumor regressions and increased therapeutic window in preclinical studies. Mol Cancer Ther. (2015) 14:A203. doi: 10.1158/1535-7163.TARG-15-A203, PMID: 41680580

[33] Wu L Seung E Xu L Rao E Lord DM Wei RR . Trispecific antibodies enhance the therapeutic efficacy of tumor-directed T cells through T cell receptor co-stimulation. Nat Cancer. (2020) 1:86–98. doi: 10.1038/s43018-019-0004-z, PMID: 35121834

[34] Seung E Xing Z Wu L Rao E Cortez-Retamozo V Ospina B . A trispecific antibody targeting HER2 and T cells inhibits breast cancer growth via CD4 cells. Nature. (2022) 603:328–34. doi: 10.1038/s41586-022-04439-0, PMID: 35197632

[35] Bacac M Fauti T Sam J Colombetti S Weinzierl T Ouaret D . A novel carcinoembryonic antigen T-cell bispecific antibody (CEA TCB) for the treatment of solid tumors. Clin Cancer Res. (2016) 22:3286–97. doi: 10.1158/1078-0432.Ccr-15-1696, PMID: 26861458

[36] Baliga R Li K Manlusoc M Hinton P Ng D Tran M . High avidity IgM-based CD20xCD3 bispecific antibody (IGM-2323) for enhanced T-cell dependent killing with minimal cytokine release. Blood. (2019) 134:1574. doi: 10.1182/blood-2019-131650, PMID: 41496790

[37] Carretero-Iglesia L Hall OJ Berret J Pais D Estoppey C Chimen M . ISB 2001 trispecific T cell engager shows strong tumor cytotoxicity and overcomes immune escape mechanisms of multiple myeloma cells. Nat Cancer. (2024) 5:1494–514. doi: 10.1038/s43018-024-00821-1, PMID: 39261676 PMC11505469

[38] Roskopf CC Braciak TA Fenn NC Kobold S Fey GH Hopfner KP . Dual-targeting triplebody 33-3–19 mediates selective lysis of biphenotypic CD19^+^ CD33^+^ leukemia cells. Oncotarget. (2016) 7:22579–89. doi: 10.18632/oncotarget.8022, PMID: 26981773 PMC5008383

[39] Shen Y Jin SJ Chen YC Liu WH Li YM Zhao WY . Improving the tumor selectivity of T cell engagers by logic-gated dual tumor-targeting. Pharmacol Res. (2023) 192:106781–90. doi: 10.1016/j.phrs.2023.106781, PMID: 37119880

[40] Tapia-Galisteo A Sánchez Rodríguez Í Aguilar-Sopeña O Harwood SL Narbona J Ferreras Gutierrez M . Trispecific T-cell engagers for dual tumor-targeting of colorectal cancer. Oncoimmunology. (2022) 11:2034355–68. doi: 10.1080/2162402x.2022.2034355, PMID: 35154908 PMC8837253

[41] Zhang M Lam K-P Xu S . Natural Killer Cell Engagers (NKCEs): A new frontier in cancer immunotherapy. Front Immunol. (2023) 14:1207276. doi: 10.3389/fimmu.2023.1207276, PMID: 37638058 PMC10450036

[42] Pandey JP Namboodiri AM . Genetic variants of IgG1 antibodies and FcγRIIIa receptors influence the magnitude of antibody-dependent cell-mediated cytotoxicity against prostate cancer cells. Oncoimmunology. (2014) 3:e27317. doi: 10.4161/onci.27317, PMID: 24701371 PMC3961482

[43] Cózar B Greppi M Carpentier S Narni-Mancinelli E Chiossone L Vivier E . Tumor-infiltrating natural killer cells. Cancer Discov. (2021) 11:34–44. doi: 10.1158/2159-8290.Cd-20-0655, PMID: 33277307 PMC7611243

[44] Bartlett NL Herrera AF Domingo-Domenech E Mehta A Forero-Torres A Garcia-Sanz R . A phase 1b study of AFM13 in combination with pembrolizumab in patients with relapsed or refractory Hodgkin lymphoma. Blood. (2020) 136:2401–9. doi: 10.1182/blood.2019004701, PMID: 32730586 PMC7685206

[45] El-Khoueiry AB Lopez JS Saavedra O Awad M Thomas J Tiu C . A phase I/IIa dose escalation study of AFM24 in patients with epidermal growth factor receptor-expressing (EGFR) solid tumors: Results from phase I. Ann Oncol. (2022) 33:S889. doi: 10.1016/j.annonc.2022.07.880, PMID: 41727822

[46] Wu J Mishra HK Walcheck B . Role of ADAM17 as a regulatory checkpoint of CD16A in NK cells and as a potential target for cancer immunotherapy. J Leukoc Biol. (2019) 105:1297–303. doi: 10.1002/jlb.2mr1218-501r, PMID: 30786043 PMC6792391

[47] Whalen KA Rakhra K Mehta NK Steinle A Michaelson JS Baeuerle PA . Engaging natural killer cells for cancer therapy via NKG2D, CD16A and other receptors. MAbs. (2023) 15:2208697–711. doi: 10.1080/19420862.2023.2208697, PMID: 37165468 PMC10173799

[48] Wensveen FM Jelenčić V Polić B . NKG2D: A master regulator of immune cell responsiveness. Front Immunol. (2018) 9:441. doi: 10.3389/fimmu.2018.00441, PMID: 29568297 PMC5852076

[49] Chitadze G Bhat J Lettau M Janssen O Kabelitz D . Generation of soluble NKG2D ligands: Proteolytic cleavage, exosome secretion and functional implications. Scand J Immunol. (2013) 78:120–9. doi: 10.1111/sji.12072, PMID: 23679194

[50] Gauthier L Morel A Anceriz N Rossi B Blanchard-Alvarez A Grondin G . Multifunctional natural killer cell engagers targeting NKp46 trigger protective tumor immunity. Cell. (2019) 177:1701–13.e16. doi: 10.1016/j.cell.2019.04.041, PMID: 31155232

[51] Pekar L Klausz K Busch M Valldorf B Kolmar H Wesch D . Affinity maturation of B7-H6 translates into enhanced NK cell-mediated tumor cell lysis and improved proinflammatory cytokine release of bispecific immunoligands via NKp30 engagement. J Immunol. (2021) 206:225–36. doi: 10.4049/jimmunol.2001004, PMID: 33268483 PMC7750860

[52] Xiao X Cheng Y Zheng X Fang Y Zhang Y Sun R . Bispecific NK-cell engager targeting BCMA elicits stronger antitumor effects and produces less proinflammatory cytokines than T-cell engager. Front Immunol. (2023) 14:1113303. doi: 10.3389/fimmu.2023.1113303, PMID: 37114050 PMC10126364

[53] Vallera DA Ferrone S Kodal B Hinderlie P Bendzick L Ettestad B . NK-cell-mediated targeting of various solid tumors using a B7-H3 tri-specific killer engager *in vitro* and in *vivo*. Cancers (Basel). (2020) 12:2659–76. doi: 10.3390/cancers12092659, PMID: 32961861 PMC7564091

[54] Felices M Warlick E Juckett M Weisdorf D Vallera D Miller S . GTB-3550 tri-specific killer engager TriKETM drives NK cells expansion and cytotoxicity in acute myeloid leukemia (AML) and myelodysplastic syndromes (MDS) patients. J Immunother Cancer. (2021) 9:A473. doi: 10.1136/jitc-2021-SITC2021.444, PMID: 41686241

[55] Demaria O Gauthier L Vetizou M Blanchard Alvarez A Vagne C Habif G . Antitumor immunity induced by antibody-based natural killer cell engager therapeutics armed with not-alpha IL-2 variant. Cell Rep Med. (2022) 3:100783–807. doi: 10.1016/j.xcrm.2022.100783, PMID: 36260981 PMC9589122

[56] Overdijk MB Verploegen S Bögels M van Egmond M Lammerts van Bueren JJ Mutis T . Antibody-mediated phagocytosis contributes to the anti-tumor activity of the therapeutic antibody daratumumab in lymphoma and multiple myeloma. MAbs. (2015) 7:311–21. doi: 10.1080/19420862.2015.1007813, PMID: 25760767 PMC4622648

[57] VanDerMeid KR Elliott MR Baran AM Barr PM Chu CC Zent CS . Cellular cytotoxicity of next-generation CD20 monoclonal antibodies. Cancer Immunol Res. (2018) 6:1150–60. doi: 10.1158/2326-6066.Cir-18-0319, PMID: 30089638

[58] Hogarth PM Pietersz GA . Fc receptor-targeted therapies for the treatment of inflammation, cancer and beyond. Nat Rev Drug Discov. (2012) 11:311–31. doi: 10.1038/nrd2909, PMID: 22460124

[59] Valerius T Repp R de Wit TP Berthold S Platzer E Kalden JR . Involvement of the high-affinity receptor for IgG (FcγRI; CD64) in enhanced tumor cell cytotoxicity of neutrophils during granulocyte colony-stimulating factor therapy. Blood. (1993) 82:931–9. doi: 10.1182/blood.V82.3.931.931, PMID: 7687898

[60] Sewnath CA Behrens LM van Egmond M . Targeting myeloid cells with bispecific antibodies as novel immunotherapies of cancer. Expert Opin Biol Ther. (2022) 22:983–95. doi: 10.1080/14712598.2022.2098675, PMID: 35854649

[61] Xu L Li B Pi C Zhu Z Tao F Xie K . Targeting CD89 on tumor-associated macrophages overcomes resistance to immune checkpoint blockade. J Immunother Cancer. (2022) 10:e005447. doi: 10.1136/jitc-2022-005447, PMID: 36460336 PMC9723960

[62] Li B Xu L Pi C Yin Y Xie K Tao F . CD89-mediated recruitment of macrophages via a bispecific antibody enhances anti-tumor efficacy. Oncoimmunology. (2017) 7:e1380142. doi: 10.1080/2162402x.2017.1380142, PMID: 29296544 PMC5739557

[63] Ye S Cohen D Belmar NA Choi D Tan SS Sho M . A bispecific molecule targeting CD40 and tumor antigen mesothelin enhances tumor-specific immunity. Cancer Immunol Res. (2019) 7:1864–75. doi: 10.1158/2326-6066.Cir-18-0805, PMID: 31462409

[64] Hägerbrand K Varas L Deronic A Nyesiga B Sundstedt A Ljung L . Bispecific antibodies targeting CD40 and tumor-associated antigens promote cross-priming of T cells resulting in an antitumor response superior to monospecific antibodies. J Immunother Cancer. (2022) 10:e005018. doi: 10.1136/jitc-2022-005018, PMID: 36323431 PMC9660648

[65] Pastan I Hassan R . Discovery of mesothelin and exploiting it as a target for immunotherapy. Cancer Res. (2014) 74:2907–12. doi: 10.1158/0008-5472.Can-14-0337, PMID: 24824231 PMC4062095

[66] Zagorulya M Spranger S . Once upon a prime: DCs shape cancer immunity. Trends Cancer. (2023) 9:172–84. doi: 10.1016/j.trecan.2022.10.006, PMID: 36357313 PMC10827483

[67] Bhandarkar V Dinter T Spranger S . Architects of immunity: How dendritic cells shape CD8^+^ T cell fate in cancer. Sci Immunol. (2025) 10:eadf4726. doi: 10.1126/sciimmunol.adf4726, PMID: 39823318 PMC11970844

[68] Houtenbos I Santegoets S Westers TM Waisfisz Q Kipriyanov S Denkers F . The novel bispecific diabody αCD40/αCD28 strengthens leukaemic dendritic cell-induced T-cell reactivity. Br J Haematol. (2008) 142:273–83. doi: 10.1111/j.1365-2141.2008.06990.x, PMID: 18492117

[69] Muik A Adams 3HC Gieseke F Altintas I Schoedel KB Blum JM . DuoBody-CD40x4-1BB induces dendritic-cell maturation and enhances T-cell activation through conditional CD40 and 4-1BB agonist activity. J Immunother Cancer. (2022) 10:e004322. doi: 10.1136/jitc-2021-004322, PMID: 35688554 PMC9189854

[70] Liu L Chen J Bae J Li H Sun Z Moore C . Rejuvenation of tumour-specific T cells through bispecific antibodies targeting PD-L1 on dendritic cells. Nat BioMed Eng. (2021) 5:1261–73. doi: 10.1038/s41551-021-00800-2, PMID: 34725504 PMC9499378

[71] Sung E Ko M Won JY Jo Y Park E Kim H . LAG-3xPD-L1 bispecific antibody potentiates antitumor responses of T cells through dendritic cell activation. Mol Ther. (2022) 30:2800–16. doi: 10.1016/j.ymthe.2022.05.003, PMID: 35526096 PMC9372323

[72] Shapir Itai Y Barboy O Salomon R Bercovich A Xie K Winter E . Bispecific dendritic-T cell engager potentiates anti-tumor immunity. Cell. (2024) 187:375–89.e18. doi: 10.1016/j.cell.2023.12.011, PMID: 38242085

[73] Rosenberg J Huang J . CD8^+^ T cells and NK cells: Prallel and complementary soldiers of immunotherapy. Curr Opin Chem Eng. (2018) 19:9–20. doi: 10.1016/j.coche.2017.11.006, PMID: 29623254 PMC5880541

[74] Ye QN Zhu L Liang J Zhao DK Tian TY Fan YN . Orchestrating NK and T cells via tri-specific nano-antibodies for synergistic antitumor immunity. Nat Commun. (2024) 15:6211–26. doi: 10.1038/s41467-024-50474-y, PMID: 39043643 PMC11266419

[75] Fan YN Zhu L Qing YX Ye SY Ye QN Huang XY . Engineering multi-specific nano-antibodies for cancer immunotherapy. Nat BioMed Eng. (2025) 9:2124–40. doi: 10.1038/s41551-025-01425-5, PMID: 40571759

[76] Lameris R Ruben JM Iglesias-Guimarais V de Jong M Veth M van de Bovenkamp FS . A bispecific T cell engager recruits both type 1 NKT and Vγ9Vδ2-T cells for the treatment of CD1d-expressing hematological Malignancies. Cell Rep Med. (2023) 4:100961–84. doi: 10.1016/j.xcrm.2023.100961, PMID: 36868236 PMC10040383

[77] Yang X Lin J Wang G Xu D . Targeting proliferating tumor-infiltrating macrophages facilitates spatial redistribution of CD8^+^ T cells in pancreatic cancer. Cancers (Basel). (2022) 14:1474–92. doi: 10.3390/cancers14061474, PMID: 35326625 PMC8946118

[78] Vermare A Guérin MV Peranzoni E Bercovici N . Dynamic CD8^+^ T cell cooperation with macrophages and monocytes for successful cancer immunotherapy. Cancers (Basel). (2022) 14:3546–58. doi: 10.3390/cancers14143546, PMID: 35884605 PMC9318008

[79] Scott EM Jacobus EJ Lyons B Frost S Freedman JD Dyer A . Bi- and tri-valent T cell engagers deplete tumour-associated macrophages in cancer patient samples. J Immunother Cancer. (2019) 7:320–37. doi: 10.1186/s40425-019-0807-6, PMID: 31753017 PMC6873687

[80] von Locquenghien M Zwicky P Xie K Jaitin DA Sheban F Yalin A . Macrophage-targeted immunocytokine leverages myeloid, T, and NK cell synergy for cancer immunotherapy. Cell. (2025) 188:7099–117.e26. doi: 10.1016/j.cell.2025.10.030, PMID: 41265436

[81] Son W Lee Y Park Y Park KS Kim S Youn H . Fc-competent TIGITx4-1BB bispecific antibody exerts potent long-lasting antitumor activity by potentiating CD8^+^ T cell activity and Fcγ receptor-mediated modulation of the tumor microenvironment. J Immunother Cancer. (2025) 13:e010728. doi: 10.1136/jitc-2024-010728, PMID: 40010766 PMC12083285

[82] Brinkmann U Kontermann RE . The making of bispecific antibodies. MAbs. (2017) 9:182–212. doi: 10.1080/19420862.2016.1268307, PMID: 28071970 PMC5297537

[83] Ridgway JB Presta LG Carter P . ‘Knobs-into-holes’ engineering of antibody CH3 domains for heavy chain heterodimerization. Protein Eng. (1996) 9:617–21. doi: 10.1093/protein/9.7.617, PMID: 8844834

[84] Sustmann C Dickopf S Regula JT Kettenberger H Mølhøj M Gassner C . DuoMab: a novel CrossMab-based IgG-derived antibody format for enhanced antibody-dependent cell-mediated cytotoxicity. MAbs. (2019) 11:1402–14. doi: 10.1080/19420862.2019.1661736, PMID: 31526159 PMC6816436

[85] Klein C Schaefer W Regula JT . The use of CrossMAb technology for the generation of bi- and multispecific antibodies. MAbs. (2016) 8:1010–20. doi: 10.1080/19420862.2016.1197457, PMID: 27285945 PMC4968094

[86] Wu X Yuan R Bacica M Demarest SJ . Generation of orthogonal Fab-based trispecific antibody formats. Protein Eng Des Sel. (2018) 31:249–56. doi: 10.1093/protein/gzy007, PMID: 29718394

[87] DiGiammarino E Ghayur T Liu J . Design and generation of DVD-Ig™ molecules for dual-specific targeting. Methods Mol Biol. (2012) 899:145–56. doi: 10.1007/978-1-61779-921-1_9, PMID: 22735951

[88] Orcutt KD Ackerman ME Cieslewicz M Quiroz E Slusarczyk AL Frangioni JV . A modular IgG-scFv bispecific antibody topology. Protein Eng Des Sel. (2010) 23:221–8. doi: 10.1093/protein/gzp077, PMID: 20019028 PMC2841541

[89] Seifert O Rau A Beha N Richter F Kontermann RE . Diabody-Ig: A novel platform for the generation of multivalent and multispecific antibody molecules. MAbs. (2019) 11:919–29. doi: 10.1080/19420862.2019.1603024, PMID: 30951400 PMC6601561

[90] Moore PA Zhang W Rainey GJ Burke S Li H Huang L . Application of dual affinity retargeting molecules to achieve optimal redirected T-cell killing of B-cell lymphoma. Blood. (2011) 117:4542–51. doi: 10.1182/blood-2010-09-306449, PMID: 21300981

[91] Weichel M Ellwanger K Fucek I Knackmuss SHJ Rajkovic E Reusch U . TandAbs: Potent and well-manufacturable bi-specific antibodies. Eur Pharm Rev. (2015) 20:27–32. doi: 10.1046/j.1365-2583.2001.00236.x, PMID: 11240633

[92] Ma J Mo Y Tang M Shen J Qi Y Zhao W . Bispecific antibodies: From research to clinical application. Front Immunol. (2021) 12:626616. doi: 10.3389/fimmu.2021.626616, PMID: 34025638 PMC8131538

[93] Chang CH Rossi EA Goldenberg DM . The dock and lock method: A novel platform technology for building multivalent, multifunctional structures of defined composition with retained bioactivity. Clin Cancer Res. (2007) 13:5586s–91s. doi: 10.1158/1078-0432.Ccr-07-1217, PMID: 17875793

[94] Yuan H Jiang W von Roemeling CA Qie Y Liu X Chen Y . Multivalent bi-specific nanobioconjugate engager for targeted cancer immunotherapy. Nat Nanotechnol. (2017) 12:763–9. doi: 10.1038/nnano.2017.69, PMID: 28459470

[95] Guan P Jin F Zhang A Gao S Liu Z . Rationally engineered bispecific nanoimmunoblocker restores anticancer immunity via dual immune checkpoint blockade. ACS Nano. (2025) 19:5392–405. doi: 10.1021/acsnano.4c13463, PMID: 39887132

[96] Li L Wang B Li Q Zhang L Li C Jin A . A TCR nanovesicle antibody for redirecting T cells and reversing immunosuppression as a tumor immunotherapy strategy. J Control Release. (2025) 384:113869–80. doi: 10.1016/j.jconrel.2025.113869, PMID: 40412660

[97] Thoreau F Chudasama V . Enabling the next steps in cancer immunotherapy: From antibody-based bispecifics to multispecifics, with an evolving role for bioconjugation chemistry. RSC Chem Biol. (2022) 3:140–69. doi: 10.1039/d1cb00082a, PMID: 35360884 PMC8826860

[98] Kim CH Axup JY Dubrovska A Kazane SA Hutchins BA Wold ED . Synthesis of bispecific antibodies using genetically encoded unnatural amino acids. J Am Chem Soc. (2012) 134:9918–21. doi: 10.1021/ja303904e, PMID: 22642368 PMC4299457

[99] Wagner K Kwakkenbos MJ Claassen YB Maijoor K Böhne M van der Sluijs KF . Bispecific antibody generated with sortase and click chemistry has broad antiinfluenza virus activity. Proc Natl Acad Sci U S A. (2014) 111:16820–5. doi: 10.1073/pnas.1408605111, PMID: 25385586 PMC4250106

[100] Thoreau F Szijj PA Greene MK Rochet LNC Thanasi IA Blayney JK . Modular chemical construction of IgG-like mono- and bispecific synthetic antibodies (SynAbs). ACS Cent Sci. (2023) 9:476–87. doi: 10.1021/acscentsci.2c01437, PMID: 36968530 PMC10037451

[101] Szijj PA Gray MA Ribi MK Bahou C Nogueira JCF Bertozzi CR . Chemical generation of checkpoint inhibitory T cell engagers for the treatment of cancer. Nat Chem. (2023) 15:1636–47. doi: 10.1038/s41557-023-01280-4, PMID: 37488375 PMC10624612

